# Consumption of sugar-sweetened beverages and fruit juice and human cancer: a systematic review and dose-response meta-analysis of observational studies

**DOI:** 10.7150/jca.51322

**Published:** 2021-03-21

**Authors:** Yuting Li, Li-liangzi Guo, Kaiyin He, Changbing Huang, Shaohui Tang

**Affiliations:** 1 Department of Gastroenterology, The First Affiliated Hospital, Jinan University, Guangzhou, Guangdong, China.; 2 Department of Information, Affiliated Hospital (Clinical College) of Xiangnan University, Chenzhou, Hunan, China.

**Keywords:** sugary beverages, cancer, risk, mortality, meta-analysis

## Abstract

**Background:** Several epidemiological studies have assessed the association of sugary drinks consumption with cancer, but the results remain controversial.

**Objective:** We performed this analysis to evaluate possible causal relationship between sugary drinks consumption and cancer risk and mortality.

**Methods:** We searched PubMed, Embase, and Web of Science databases in English. Observational studies evaluating the association of sugary drinks intake with cancer were included. Random-effects meta-analysis was used to calculate the risk estimates.

**Results:** A total of 71 observational articles with 32 case-control and 39 cohort studies were included in the meta-analysis. 60 addressed cancer risk, and 11 reported cancer mortality. Compared with the lowest level, the highest level of sugar-sweetened beverages (SSB) consumption showed an increased overall cancer risk (RR=1.12 95% CI: 1.06-1.19, P=0.000) and mortality (RR=1.07 95% CI: 1.01-1.14, P=0.029), and fruit juice intake showed a positive association with cancer risk in cohort studies (RR=1.06 95% CI: 1.01-1.11, P=0.013). Subgroup analyses based on cancer type indicated that risk of breast cancer, hepatocellular carcinoma, colorectal cancer, and prostatic cancer mortality had a positive association with SSB consumption. For dose-response analysis, evidence of a linear association was found between overall cancer risk and SSB or fruit juice consumption, and the risk increase by 4% for one servings/d increment in SSB intake and 14% in fruit juice.

**Conclusions:** Our findings suggest the consumption of sugary beverages may increase the risk and mortality of cancer, especially risk of breast cancer, hepatocellular carcinoma, colorectal cancer, and prostatic cancer, and mortality of breast cancer, though the evidence was limited.

## Introduction

Cancer is one of the leading causes of morbidity and mortality worldwide, which responsible for nearly 18.1 million new cases and 9.6 million deaths in 2018 1. It is well-known that is a complicated disease caused by interaction of genetic and environmental factors, such as smoking, physical exercise, and diet 2-4. In terms of diet, some food such as red and processed meat may increase cancer risk and mortality 5, 6, and some such as vegetables seem to decrease cancer incidence 7 and mortality 8. However, the association between sugary drinks and cancer is still uncertain.

The consumption of sugary beverages (such as sugary-sweetened beverages (SSB) and fruit juice), has increased all over the world in the last decades, especially in adolescents 9. SSB, including soft drink, carbonated drinks, artificially sweetened drinks and fruit drinks (lemonade and punch), are defined as beverages containing added caloric sweetener (sucrose, fructose, artificial sweetener, etc.). Investigations of sugary beverages and its potential health effects have been an active area of research interest. Several studies have found that sugary drinks is associated with having a higher risk of type 2 diabetes 10, hypertension and cardiometabolic disease 11, as well as a greater risk of depression 12 and non-alcoholic fatty liver disease 13. However, the potential link between sugary drinks and cancer is inconsistent, with some reporting a positive association 14, some a negative association 15 and others finding no relationship 16.

To better understand the relationship between the consumption of sugary beverages (SSB and fruit juice) and cancer risk and mortality, we combined all published epidemiologic studies on this issue and conducted a dose-response meta-analysis.

## Methods

Following the Preferred Reporting Items for Systematic reviews and Meta-Analysis (PRISMA) guidelines 17, we performed a meta-analysis and systematic review dealing with the association of sugary beverages with cancer risk and mortality in human. We employed the PICO format (population, intervention, comparison, outcome) to answer the research question: “Are sugary beverages consumption (SSB and fruit juice) associated with cancer risk and mortality. Population: Adults with any type of cancer; Intervention: SSB and fruit juice; Comparison: Adults without cancer; O: The risk and mortality of cancer.

### Search strategy

There is a two-step search strategy. First, a systematic review was performed by querying Pubmed, Embase and Web of science database in English until October 2020. The keywords we used as “sugary beverages or sweetened beverages or fruit juice or soft drink or carbonated drink or soda beverages” combined with “cancer or tumor or carcinoma or melanoma or sarcoma or neoplasms or lymphoma or leukemia”. In the second part, we searched the bibliographies of retrieved publications to further increase the yield of potentially relevant articles. For studies that did not report outcomes of interest, we contacted the authors via email. Two independent reviewers (Y.T.L and K.Y.H) made an initial judgment of whether the studies were eligible to be included in the analysis, and any disagreements were resolved by consulting the third investigator (S.H.T).

### Inclusion and exclusion criteria

The inclusion criteria were required as follows: (1) adult participants; (2) observational studies that investigated sugar-sweetened beverages/fruit juice consumption and cancer risk and mortality. SSB included regular sugar-sweetened soda, soft drinks, artificially sweetened drinks and fruit drinks (such as lemonade and punch). Fruit juice included apple juice, orange juice, grapefruit juice and other juice (without vegetable juice); (3) Studies reported the risk estimates (hazard ratio (HR) or relative risk (RR) or Odds ratio (OR)) with their corresponding 95% confidence interval (CI) or original data allowing us to compute them were available; (4) if the published studies reported data for specific subgroups, results for the whole population were considered in this meta-analysis; (5) if the original publications provided several independent studies, they were considered as separate studies in the following data analysis.

Exclusion criteria: (1) animal studies; (2) pregnancy women; (3) did not provide enough data on sugary beverage consumption and cancer risk or mortality; (4) duplicate reports, abstracts and review articles.

### Data extraction and quality assessment

Data extraction from each study included the name of the first author, study design, publication year, study region, sample size (number of cases and total number of participants), type of cancer, sugary beverages categories, the RRs with their 95% CIs for each category of sugary beverages intake and covariates adjusted for in the multivariable analysis. We extracted the RRs that reflected the greatest degree of adjustment for potential confounders. Two investigators (L.L.Z.G and C.B.H) independently extracted the data, and discrepancies were resolved through consensus.

The methodological quality of included studies was evaluated based on the Newcastle-Ottawa Scale (NOS) 18 for assessing the quality of observational studies in meta-analysis. A star system of the NOS ranges from 0 to 9 and contains eight questions grouped under three categories: selection, comparability, and exposure. The score of 7 or higher in case-control studies and cohort studies was considered as the high-quality studies. Study quality was assessed independently by two of the investigators (L.L.Z.G and K.Y.H), and any discrepancies were addressed by a joint reevaluation of the original article.

### Statistical analysis

The results were expressed in terms of RR and 95% CI for the highest versus the lowest category of sugary beverages consumption. Dose-response analysis were also conducted and P<0.05 was considered statistically significant. To assess the heterogeneity in results of individual studies, Cochran's Q-test and *I^2^* statistics were used. And *I^2^* >50% and P<0.1 was considered as statistically significant heterogeneity 19. The causes of heterogeneity were investigated by subgroup analysis based on study design, cancer types, geographic location, patient sex, and number of cases, study quality score, and type of food frequency questionnaire (FFQ), if data permitted. And, to examine the stability of results, sensitivity analysis by omitting one study at a time and recalculating the pooled RR was also performed. Effect differences were formally tested by means of random effects meta-analysis. According to the Cochrane Handbook, if ≥10 studies are available, publication bias was evaluated with the Begg's test and Egger's test. When P<0.05, publication bias exists. To reduce the potential influence of publication bias, we used the trim and fill method 20.

Moreover, a dose-response meta-analysis was carried out in ≥2 prospective cohort studies to assess the trend between different exposure levels of sugary drinks and cancer incidence and mortality using a random effects meta-regression 21. The dose-response relation analysis was estimated using the two-stage generalized least squares trend estimation 21. This method requires that the distribution of cases and person-years or non-cases and RRs with 95% CI for at least three categories of exposure to sugary beverages. The generalized least-squares trend and variance-weighted least squares methods require median values for categories of intake levels. When medians and means were not presented, the category mid-point was used. If the highest category was open ended, we assumed the size of the open-ended interval to the same as that of the closest interval. The consumption of sugary drinks was used to assess the exposure levels in different studies, so the intake in one serving/weeks was estimated in the dose-response analysis. Doses reported as cups or glasses per day or per month were transformed into servings/w. The results in the forest plots are presented for every seven servings/w (one serving/day) increment in sugary drinks consumption. All statistical analysis was performed using STATA version 12.0.

## Results

### Search results and study characteristics

**Figure 1:**
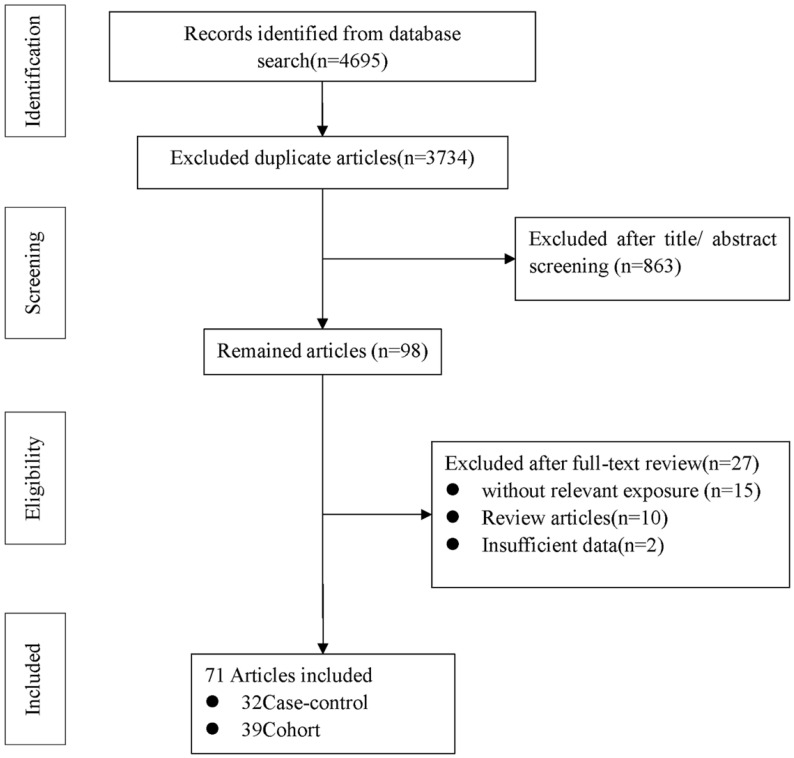
Flow diagram of literature search and study selection.

**Figure 1** shows the flow diagram of detailed selection process. A total of 4695 potentially relevant articles were initially retrieved, then 3734 duplicate articles were excluded. After screening the title and assessing the abstract, 98 articles were remained for full text review. Among them, 27 articles were excluded (10 were review articles, 15 were unmatched with the study exposure, and 2 did not provide insufficient data). In the end, a total of 71 eligible articles 14-16, 22-78 were included in our meta-analysis: 32 case-control 14-16, 22, 24-28, 30, 36, 40, 42-47, 51, 52, 54, 55, 62-64, 67, 70, 73, 75-77 and 39 cohort studies 23, 29, 31-35, 37-39, 41, 48-50, 53, 56-61, 65, 66, 68, 69, 71, 72, 74, 78 (Supplementary Table S1).

A total of 60 included articles to investigate the association between sugary drinks and cancer risk had 50122 cases with cancers originating from the following 16 sites involved in 2 hepatocellular carcinoma (HCC) 67, 74, 10 colorectal cancer 22, 24, 29, 35, 38, 52, 53, 61, 76, 78, 6 gastric cancer 14, 16, 38, 55, 64, 66, 2 lymphomas 56, 71, 8 breast cancer 28, 29, 34, 38, 53, 54, 63, 68, 8 prostatic cancer 29, 31, 33, 38, 42, 53, 57, 73, 4 renal cancer 40, 50, 65, 75, 6 bladder cancer 25, 30, 43, 44, 69, 77, 1 leukemia 71, 5 esophageal cancer 15, 16, 47, 55, 66, 2 biliary tract cancer 49, 74, 3 endometrial cancer 38, 41, 45, 11 pancreatic cancer 23, 26, 27, 36, 37, 48, 51, 58-60, 72, 2 ovary cancer 38, 46, 3 nasopharyngeal cancer 62, 66, 70 and 2 glioma 32, 39. Among them, 53 eligible studies were included to investigate the association between SSB and cancer risk, and 17 studies to evaluate the association between fruit juice and cancer risk. Of the studies, 23 14, 24, 25, 29, 31, 36, 37, 43, 47-49, 59, 60, 62, 65, 67-70, 74-77 were conducted in Europe, 29 16, 23, 26-28, 32-35, 39-42, 44-46, 50, 51, 53, 55-57, 61, 63, 66, 71-73, 78 in North America, 3 54, 58, 64 in Asia, 3 15, 22, 38 in Oceania, 1 30 in South America, 1 52 in Africa. The main characteristics of the included studies are illustrated in Supplementary Table S1.

A total of 11 eligible articles 79-89 (10 cohorts) involved in 7 overall cancer 79, 83, 84, 86-89, 3 colorectal, and 1 upper aerodigestive tract 85 were studied to review the association of SSB with cancer mortality. Of the studies, 1 86 were conducted in Europe, 3 83, 87, 89 in Asia, and 7 79-82, 84, 85, 88 in North America. No enough study was provided to evaluate the association between fruit juice and cancer mortality.

The quality on the basis of the NOS score was described in Supplementary Tables S1. NOS scores ranged from 5 to 9, and study quality was maximal (nine stars) in cases (n=5); lower quality studies were graded with eight stars (n=19), seven stars (n=19), six stars (n=19), and five stars (n=9).

### Sugar-sweetened beverages (SSB) and cancer risk

#### Highest vs lowest category meta-analysis

**Figure 2:**
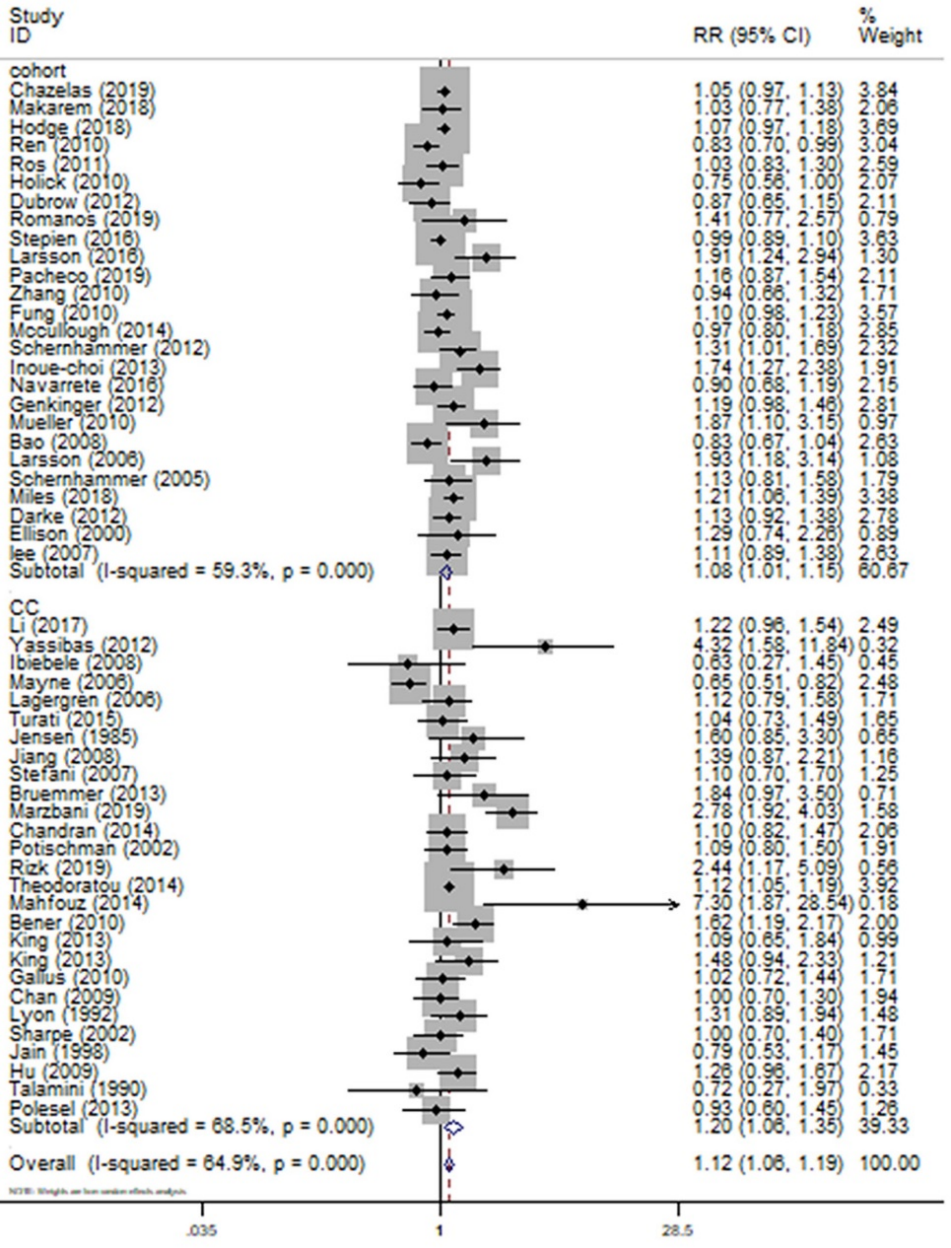
Meta-analysis of SSB consumption and cancer risk by study design. Forest plot showing the summary relative ratio (RR). Weights are from random-effects analysis.

For the primary outcome of cancer incidence, a total of 53 14-16, 23-25, 27-33, 35-59, 61-63, 66-69, 71-78 articles including 26 23, 29, 31-33, 35, 37-39, 41, 48-50, 53, 56-59, 61, 66, 68, 69, 71, 72, 74, 78 cohort studies and 27 14-16, 24, 25, 27, 28, 30, 36, 40, 42-47, 51, 52, 55, 62, 63, 67, 73, 75-77 case-control studies with 44370 cases were used to evaluate the pooled RR. Highest category versus lowest category of SSB consumption could have a significantly positive association with overall cancer incidence by 12% (RR=1.12 95%CI: 1.06-1.19, P=0.000; *I^2^*=64.9%) (Fig. 2). Evidence in favor of an association with cancer risk was weaker among cohort studies (RR=1.08 95%CI: 1.01-1.15, P=0.020; *I^2^*=59.3%) (Fig. 2) when compared with case-control studies (RR=1.20 95%CI: 1.06-1.35, P=0.003; *I^2^*=68.5%) (Fig. 2). Nevertheless, the heterogeneity between-study did not decrease remarkable across studies with the same design.

Table 1 shows the results of subgroup analysis by cancer type. The results showed that the greatest risk of cancer following SSB consumption was observed for breast cancer (n=7, RR=1.21 95% CI: 1.02-1.43, P=0.027; *I^2^*=62.5%), HCC (n=2, RR=2.00 95% CI: 1.33-3.03, P=0.001; *I^2^*=0%), colorectal cancer (n=9, RR=1.14 95% CI: 1.01-1.27, P=0.030; *I^2^*=66.6%), prostatic cancer (n=8, RR=1.14 95% CI: 1.05-1.24, P=0.003; *I^2^*=0%). In contrast, meta-analysis suggested no evidence of association for the following tumor site: esophageal cancer (n=5), gastric cancer (n=6), renal cancer (n=4), bladder cancer (n=6), ovary cancer (n=2), endometrial cancer (n=3), pancreatic cancer (n=9), hematopoietic cancer (n=2), nasopharyngeal cancer (n=2), biliary tract cancers (n=2). In our analysis, SSB consumption seemed to be linked to a statistical significantly lower risk of glioma (n=2, RR=0.81 95% CI: 0.66-0.99, P=0.041; *I^2^*=0%) and non-cardia gastric cancer (n=2, RR=0.69 95% CI: 0.50-0.95, P=0.022; *I^2^*=0%).

To assess the potential modifying effects, subgroup analysis by geographic location, number of cases, study quality score, and type of questionnaires was conducted for all SSB studies involved in overall cancer and each cancer (Supplementary Table S2). Overall, a positively association were observed between highest vs lowest intake of SSB and overall cancer risk in the stratified analysis by study quality score, and type of food frequency questionnaires (FFQ). By geographic location, the association was significant in European (n=19, RR=1.23 95%CI: 1.11-1.37, P=0.001; *I^2^*=58.2%) and in Asia (n=2, RR=2.39 95%CI: 1.64-3.48, P<0.001; *I^2^*=31.4%), but nonsignificant in North America (n=28, RR=1.06 95%CI: 0.66-1.45, P=0.147; *I^2^*=59%). Similarly, the association stratified by number of cases was significant in group of <500 cases (n=28, RR=1.25 95%CI: 1.12-1.41, P=0.000; *I^2^*=69.5%), but not in ≥500 cases (n=25, RR=1.06 95%CI: 0.99-1.13, P=0.076; *I^2^*=59.7%). For cohort studies, only subgroup analysis in validated FFQ and high-quality score had an increased risk of cancer incidence with RR of 1.10 (n=15) and 1.08 (n=26). For case-control studies, subgroup analysis in European, <500 cases, validated FFQ and low-quality score had an increased risk of cancer incidence with RR of 1.40 (n=12), 1.43 (n=16), 1.12(n=5), 1.25 (n=19). The rest of group in cohort and in case-control studies were not significantly associated with SSB consumption (Supplementary Table S2). For each cancer, the results of subgroup analysis were also showed in Supplementary Table S2, which suggests that the relationship between consumption of SSB and cancer risk may vary with different tumors.

### Dose-response meta-analysis

**Figure 3:**
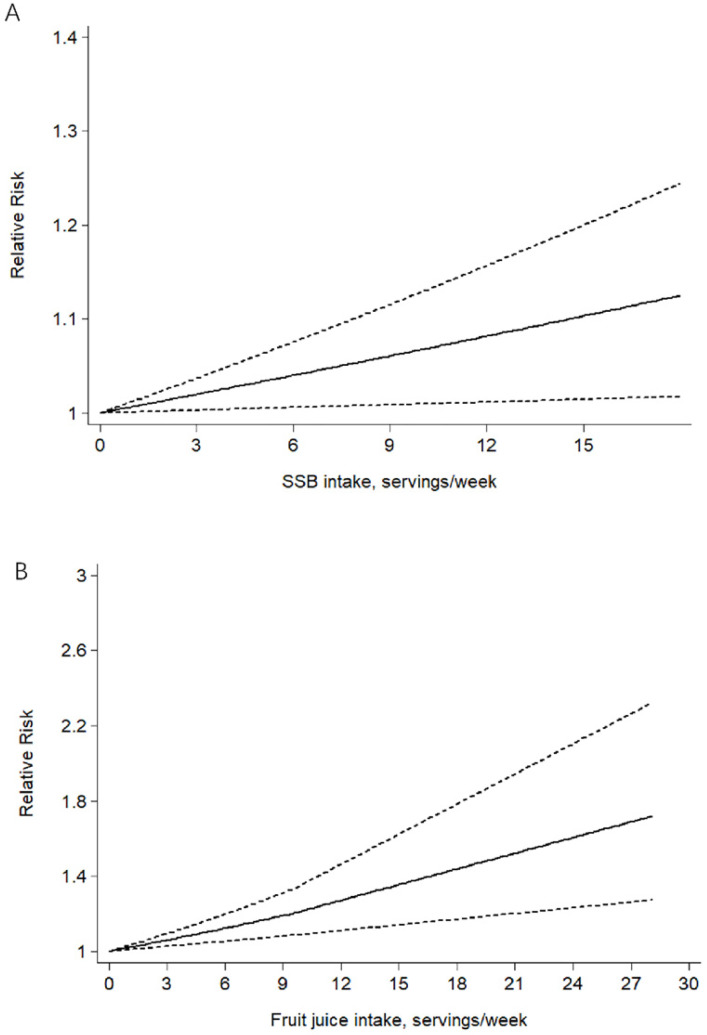
The linear dose-response association meta-analysis between SSB (A) and fruit juice (B) consumption and risks of cancer in prospective cohort studies. Weights are from random-effects analysis.

Combing data from 20 23, 29, 32, 33, 38, 39, 48-50, 53, 56-59, 61, 66, 68, 71, 72, 78 prospective cohort studies, trend meta-analysis showed a statistically significant positive dose-response relationship between SSB and overall cancer incidence from linearity (P-_nonlinearity_=0.802). We found that one servings/d increment in SSB consumption could increase 4% risk of overall cancer (RR=1.04 95%CI: 1.01-1.09, P=0.022) (Fig. 3A) using random model with significant heterogeneity. In the light of the statistically significant heterogeneity (P=0.032), we investigated its potential sources. Subgroup analysis by cancer type showed no evidence of dose-response relationship for the following tumor site: gastric cancer (n=2), breast cancer (n=4), colorectal cancer (n=5), prostatic cancer (n=5), pancreatic cancer (n=6), endometrial cancer (n=2), renal cancer (n=2), hematopoietic cancer (n=2) and glioma (n=2), with no heterogeneity.

### Sensitivity analysis and publication bias

We also conducted a sensitivity analysis to investigate the influences of single studies on the overall risk estimate by omitting one study in each turn (Supplementary Table S5). The omission of any study made no significant difference in the overall, cohort, and case-control studies meta-analysis, respectively.

For overall cancer, the Egger's test revealed evidence of publication bias across studies (Egger's *P*=0.005, Begg's *P*=0.002). However, there was a low probability of publication bias in case-control studies (Egger's *P*=0.082, Begg's *P*=0.076) and in cohort studies (Egger's *P*=0.051, Begg's *P*=0.047). The funnel plot of the studies is presented in Figure S1. According to the trim and fill method, which looks for missing studies based on a random-effects model, we found the results were not relatively stable. So, the evidence was poor to identify SSB as a risk factor for cancer incidence.

### Fruit juice and cancer risk

#### Highest vs lowest category meta-analysis

**Figure 4:**
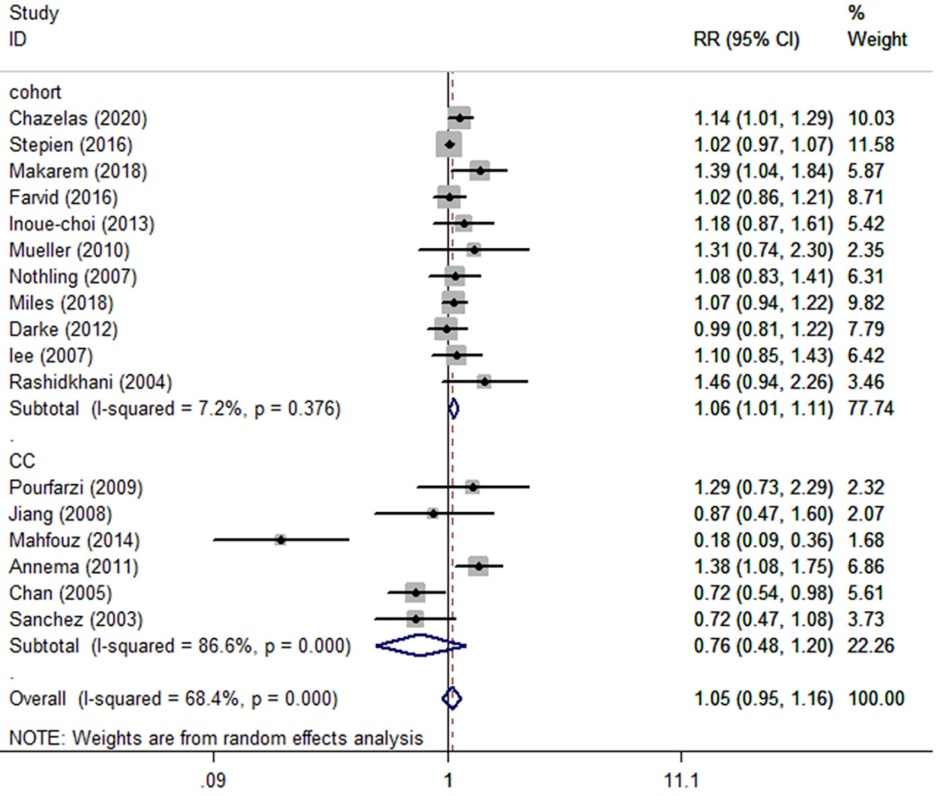
Meta-analysis of fruit juice consumption and cancer risk by study design. Forest plot showing the summary relative ratio (RR). Weights are from random-effects analysis.

Seventeen 22, 26, 29, 31, 34, 41, 44, 50, 52, 53, 57, 58, 60, 64, 65, 70, 74 published studies involved in 15192 cases reporting fruit juice consumption and cancer incidence met the inclusion criteria and were included in our meta-analysis. The pooled summary effect size indicated no significant association between fruit juice consumption and cancer incidence in overall and case-control studies. For eleven 29, 31, 34, 41, 50, 53, 57, 58, 60, 65, 74 cohort studies, however, the highest category showed a 6% increased risk of overall cancer (RR=1.06 95%CI: 1.01-1.11, P=0.013; *I^2^*=7.2%) (Fig. 4 and Table 2) compared to the lowest category.

To assess the potential modifying effects, subgroup analysis by cancer type, geographic location, number of cases, study quality score, and type of FFQ was conducted for all fruit juice studies. Subgroup analysis suggested no evidence of association for the following cancer type (Table 2): colorectal cancer (n=4), pancreatic cancer (n=3), breast cancer (n=3), and renal cancer (n=2). Except for the 11 studies with high-quality score by which the result showed a positive association between highest vs lowest intake of fruit juice and cancer incidence (RR=1.06), the other results stratified by geographic location, number of cases, and type of FFQ showed no association (Supplementary Table S3). For cohort studies, however, subgroup analysis in European, ≥500 cases, unvalidated FFQ and high-quality score had an increased risk of cancer incidence with RR of 1.14 (n=5), 1.08 (n=6), 1.11 (n=5), and 1.06 (n=11), respectively (Supplementary Table S3). For case-control studies, subgroup analysis revealed no association between fruit juice and cancer incidence.

### Dose-response meta-analysis

Combing data from 7^29^, ^34^, ^50^, ^53^, ^57^, ^58^, ^60^ prospective cohort studies, trend meta-analysis showed a statistically significant positive dose-response relationship between fruit juice and overall cancer incidence from linearity (P-_nonlinearity_=0.778). We found that one servings/d increment in fruit juice consumption could increase 14% risk of overall cancer (RR=1.14 95%CI: 1.06-1.23, P=0.000) (Fig. 3B) using random model with no heterogeneity (P=0.447). And the rest showed no evidence of dose-response relationship.

### Publication bias and sensitivity analysis

The results of Egger's test showed no evidence of publication bias for the analysis between overall cancer incidence and fruit juice consumption (Egger's *P*=0.442, Begg's *P*=0.650), but a publication bias in cohort studies (Egger's *P*=0.018, Begg's *P*=0.029). We used the trim and fill method, founding the results were not relatively stable. So, the evidence was poor to identify fruit juice as a risk factor for cancer incidence. The funnel plot of the studies is presented in Figure S2. The sensitivity analysis was conducted to investigate the influences of single studies on the overall risk estimate by omitting one study in each turn (Supplementary Table S6).

### Sugar-sweetened beverages (SSB) and cancer mortality

#### Highest vs lowest category meta-analysis

**Figure 5:**
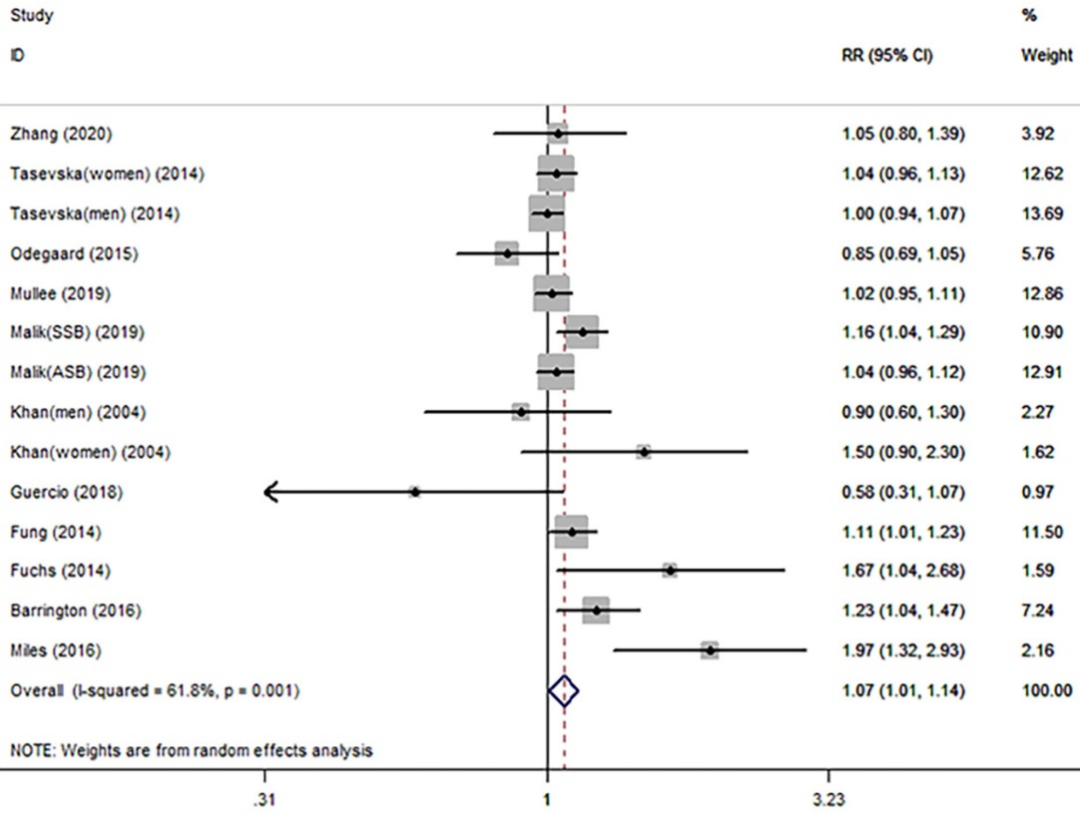
Meta-analysis of SSB consumption and cancer mortality. Forest plot showing the summary relative ratio (RR). Weights are from random-effects analysis.

For the primary outcome of cancer mortality, a total of 11 articles 79-89 involved in 10 cohort studies and 1 case-control study were included to evaluated the pooled RR. Highest category versus lowest category of SSB consumption showed a significantly positive association with overall cancer mortality (RR=1.07 95%CI: 1.01-1.14, P=0.029; *I^2^*=61.8%) (Fig. 5 and Table 3). Evidence in favor of an association with cancer mortality was the same as 10 79-84, 86-89 cohort studies (RR=1.06 95%CI: 1.00-1.12, P=0.046; *I^2^*=50.9%). Table 3 shows the results of subgroup analysis by cancer type. The results showed that only breast cancer mortality had a significant association with SSB consumption (RR=1.17 95%CI: 1.03-1.34, P=0.017; *I^2^*=0%). Colorectal cancer, prostate cancer and lung cancer seemed to be no association with SSB. Other subgroup analyses by geographic location, number of cases, study quality score, and type of FFQ were conducted for the association between overall cancer mortality and SSB consumption in Supplementary Table S4. The results in validated FFQ and North America showed an increased risk of cancer mortality with RR of 1.12 (n=5) and 1.10 (n=7), and the nonsignificant associations were observed in the other subgroup analyses.

### Dose-response meta-analysis

Combing data from 6 cohort studies 80, 82, 84, 86, 87, 89, a trend meta-analysis showed no significant dose-response relationship between SSB and overall cancer (P=0.561) or colorectal cancer (P=0.867) mortality.

### Sensitivity analysis and Publication bias

The results of Egger's test showed no evidence of publication bias for the analysis between overall cancer mortality and SSB (Egger's *P*=0.506, Begg's *P*=0.189). The funnel plot of the studies is presented in Figure S3. The sensitivity analysis was conducted to investigate the influences of single studies on the overall mortality estimate by omitting one study in each turn (Supplementary Table S7).

## Discussion

This present meta-analysis with 71 observational articles was designed to investigate the association between the consumption of sugary drinks (SSB and fruit juice) and cancer risk and mortality. To some extent, the results of this meta-analysis support the hypothesis that SSB consumption was associated with a significant increased overall cancer risk and mortality, and fruit juice intake also significantly increased overall cancer risk in cohort studies. What's more, a significant dose-response relationship was observed between SSB or fruit juice consumption and overall cancer risk, strengthening this hypothesis. When we considered cancers by site, the incidence of breast cancer, HCC, CRC, and prostatic cancer had an increased risk with SSB consumption, which was consistent with the overall results. We also found that SSB seem to play a preventive role in glioma and non-cardia gastric cancer. However, these results were based on only 2 glioma and 2 non-cardia gastric cancer studies.

The most important advantage of this meta-analysis is that, to the best of our knowledge, this is the latest, the most comprehensive and the most meaningful article. It updates and expands two previous meta-analyses. The first meta-analysis conducted by Boyle et al. 90 showed no link between the consumption of carbonated beverages and the risk of overall cancer and specific cancer without a statistical analysis, unlike our findings. We determined RRs and dose-response risk functions for the association between SSB consumption and a large number of neoplasms, some of which were never investigated using a meta-analytic approach. The second meta-analysis by Milajerdi et al. included 5 cohort studies and 4 case-control studies, and the results from both the 5 cohort and the 4 case-control studies indicated that there were no significant associations between sugary drinks consumption and PC risk (P>0.05); also, the subgroup analyses based on study location (USA/Non-USA) and follow-up duration (≥10 years/<10 years) showed that SB consumption was not associated with the risk of PC (P>0.05). In our study, we also included the aforementioned 5 cohort and 4 case-control studies and the other two studies 48, 72 to evaluate the association between SSB or fruit juice and PC**;** furthermore, we conducted the subgroup analyses by study design (cohort/case-control), geographic location (European, North America, and Asia), number of cases (≥500/<500), study quality score (>6/≤6), and type of questionnaires (FFQ/no-FFQ). All the results indicated that sugary drinks consumption was not associated with the risk of PC (P>0.05), which was consistent with the report by Milajerdi et al. 91. However, more importantly, we also evaluated the potential link between the consumption of SSB and fruit juice and overall cancer risk or mortality. Moreover, the study by Philipsborn et al. 92 assessed the effects of environmental interventions on the consumption of SSB. They focused on measures that helped people to drink fewer SSB to improve their health, but did not investigate effects of SSB on cancer risk or mortality.

In a large, high-quality prospective cohort study^90^, Chazelas et al. reported that the consumption of SSB was positively associated with the risk of overall cancer and breast cancer, and fruit juice intake was also associated with an increased risk of overall cancers, which was consistent with our main findings. In line with our results, another large prospective cohort study 38 showed that the consumption of sugar-sweetened drinks increased the risk of colorectal cancer and breast cancer. Moreover, Malik et al. 84 found that SSB consumption was associated with a higher risk of cancer mortality in a large, high-quality prospective cohort analysis, which was also consistent with our findings.

In addition, several previous studies listed some findings to describe the association between SB and human health. The report by Ferreira-Pego et al.^93^ showed consumption of >5 servings/d sugary drinks was associated with a higher risk of metabolic syndrome and hypertension; Mullee et al 86 examined a large multinational cohort of people to evaluate the association between sugary drinks and all-cause mortality, and indicated that higher all-cause mortality was found among participants who consumed ≥2 glasses/d of sugary drinks; the report by Anderson et al. 94 also showed all-cause mortality was positively associated with total SSB intake. These results were consistent with our findings.

Although it is impossible to draw causal links on the basis of these data, there are several possible explanations for the association between sugary drinks consumption and cancer. The first explanation for the increased cancer risk and mortality is that sugary drinks contain a large amount of sugar, which in part responsible for high dietary glycemic index and obesity, may lead to diabetes-related cancer (liver, prostatic, endometrium, colorectal, breast, bladder)^95^, ^96^. In addition, sugary beverages can promote insulin-glucose dysregulation, oxidative stress, inflammation, and adiposity and finally cause steroid hormone imbalances, which collectively increase cancer risk 96-98. Secondly, some chemical compounds also play an important role, such as 4-methylimidazole, an additive in drinks that contain caramel coloring (e.g., sodas) or pesticides that might be associated with increased risk of cancer and could be present in fruit juice 99, 100. Thirdly, postprandial hyperglycemia induced by diets high in sugars triggers insulin and insulin-like growth factor I synthesis, which may enhance tumor development through promoting cell proliferation and inhibiting apoptosis 88. For the decreased cancer risk including non-cardia gastric, and glioma, the current study provides no evidence that sugar-sweetened beverages consumption would be an effective strategy to lower the cancer incidence. For upper gastrointestinal tract cancer risk, they provided little support for an inverse association between sugary drinks consumption and cancer risk. Some researchers 14, 16, 101 suggested sugary drinks increase gastric reflux, and thus could be associated with an increased risk of esophageal and gastric cancer. Some 55, 66 found there is null results. For glioma risk, Dubrow et al. 32 observed a borderline-significant inverse association between glioma risk and the highest levels of intake of soda, without any dose-response relationship. So, we cannot exclude the possibility that higher sugary drinks intake than that observed in this study may be associated with an elevated risk of cancer. Further longitudinal studies are needed to shed light on this inconsistent result.

The strengths of our study include the large number of cancer cases that ensure a greater precision and high statistical power of the results. Our findings provide an assumption that sugary beverages consumption, including SSB and fruit juice, should be further considered as a risk factor for overall cancer risk and mortality. We also focused on dose-response analysis, which provide more compelling evidence to assess these associations. Even so, we have to admit that there are several limitations in our meta-analysis. Firstly, publication bias between studies not allow us to draw definitive conclusion on the role of SSB and fruit juice in the development of malignant disease in general. Studies included in the analysis were the observational studies such as case-control and cohort studies, which are more susceptible to biases, such as selection bias and recall bias. Secondly, though a sensitivity analysis showed the stability of these results by omitting one study, the disparities still lie in potential biases of each study, the definition and range of sugary beverages consumption, the type of questionnaire, and the confounders for which analysis were adjusted. These differences may all affect the accuracy of these results. Thirdly, we could not make a distinction between soft drink, carbonated drink, and artificially drink, as well as did not perform a subgroup analysis by genders. Because these projects are always mixed in some original articles. The last limitation is that the included study populations mainly come from Europe and North America, where the people have specific dietary behaviors. So, the study coverage in the world was limited because of a small amount studied from Africa, and Asia. Therefore, the overall findings of increased in cancer risk should not be overemphasized.

## Conclusions

In conclusion, our results suggest a positive relationship between the consumption of sugary beverages and overall cancer risk and mortality, though the evidences were limited. More large and precise prospective studies are required to further assess the association and the underlying mechanisms between them.

## Supplementary Material

Supplementary figures and tables.

## Figures and Tables

**Table 1:** SSB consumption and cancer risk

Factors	Number of studies	Random-effect	P value	Heterogeneity		Begg's/Egger's
		Pooled RR (95%CI)		I^2^	p	
**SSB**						
***Total***	53	**1.12 (1.06,1.19)**	**P=0.000**	64.9%	0.000	0.002/0.005
Cohort	26	**1.08 (1.01,1.15)**	**P=0.020**	59.3%	0.000	0.047/0.051
CC	27	**1.20 (1.06,1.35)**	**P=0.003**	68.5%	0.000	0.076/0.082
D-R	20	**1.04 (1.01,1.09)**	**P=0.022**		0.032	
**EC**	5	0.84 (0.63,1.12)	P=0.240	59%	0.099	
EADC	5	0.93 (0.65,1.34)	P=0.709	64.1%	0.025	
ESCC	3	0.68 (0.43,1.08)	P=0.105	41.5%	0.181	
CC	4	0.79 (0.54,1.16)	P=0.232	69.3%	0.006	
Cohort	1	0.99 (0.67,1.47)	P=0.974	0.0%	0.513	
**GC**	6	0.99 (0.79,1.29)	P=0.960	48.3%	0.043	
Cardia	5	1.03 (0.86,1.24)	P=0.717	0%	0.617	
Non-cardia	2	**0.69 (0.50,0.95)**	**P=0.022**	0%	0.668	
CC	4	1.09 (0.73,1.63)	P=0.660	68.0%	0.008	
Cohort	2	0.94 (0.73,1.22)	P=0.664	0.0%	0.637	
D-R	2	1.02 (0.97,1.06)	P=0.414		0.159	
**Breast**	7	**1.21 (1.02,1.43)**	**P=0.027**	62.5%	0.004	
Premenopausal	4	1.24 (0.96,1.61)	P=0.101	66.5%	0.011	
postmenopausal	4	1.10 (0.89,1.36)	P=0.394	49.3%	0.066	
CC	3	1.38 (0.90,2.10)	P=0.137	84.3%	0.000	
Cohort	4	1.11 (0.98,1.26)	P=0.089	0%	0.630	
D-R	4	1.09 (0.98,1.23)	P=0.116		0.144	
**HCC**	2	**2.00 (1.33,3.03)**	**P=0.001**	0%	0.526	
**BTC**	2	1.01 (0.90,1.13)	P=0.866	58.9%	0.045	
EHBC	2	1.22 (0.66,2.27)	P=0.530	79.8%	0.026	
IHBC	2	0.97 (0.90,1.05)	P=0.492	0%	0.445	
Gallbladder	2	1.30 (0.53,3.16)	P=0.566	80.8%	0.023	
Vater cancer	1	1.02 (0.95,1.10)	P=0.586	0%	0.434	
**CRC**	9	**1.14 (1.01,1.27)**	**P=0.030**	66.6%	0.000	
CC	**3**	**2.04 (1.16,3.59)**	**P=0.014**	86.9%	0.000	
Cohort	6	1.07 (0.97,1.18)	P=0.168	28.3%	0.193	
D-R	4	1.01 (0.93,1.10)	P=0.302		0.736	
**Prostatic**	8	**1.14 (1.05,1.24)**	**P=0.003**	0%	0.528	
CC	2	0.90 (0.70,1.17)	P=0.442	0%	0.380	
Cohort	6	**1.17 (1.07,1.28)**	**P=0.001**	0%	0.800	
D-R	4	1.09 (0.87,1.36)	P=0.445		0.661	
**Pancreatic**	9	1.12 (0.95,1.32)	P=0.165	57.9%	0.015	
CC	3	1.08 (0.89,1.32)	P=0.446	0%	0.526	
Cohort	6	1.16 (0.92,1.47)	P=0.217	71.7%	0.003	
D-R	6	1.11 (0.94, 1.32)	P=0.207		0.002	
**Ovarian**	2	1.24 (0.88,1.74)	P=0.225	0%	0.821	
**Endometrial**	3	1.32 (0.95,1.84)	P=0.099	45.8%	0.137	
CC	1	1.48 (0.94,2.33)	P=0.090			
Cohort	2	1.21 (0.73,1.99)	P=0.460	63.6%	0.064	
**Renal**	4	1.16 (0.99,1.36)	P=0.062	0%	0.625	
CC	2	1.18 (0.82,1.68)	P=0.372	11.6%	0.288	
Cohort	2	1.14 (0.94,1.38)	P=0.198	0%	0.511	
D-R	2	1.07 (0.92,1.24)	P=0.354		0.740	
**Bladder**	6	1.14 (0.98,1.33)	P=0.095	0%	0.515	
CC	5	**1.25 (1.01,1.54)**	**P=0.040**	0%	0.579	
Cohort	1	1.03 (0.82,1.29)	P=0.796			
**Nasopharyngeal**	2	0.81 (0.66,1.00)	P=0.051	0%	0.941	
CC	1	0.78 (0.62,0.99)	P=0.747			
Cohort	1	0.93 (0.60,1.45)	P=0.042			
**Hematopoietic**	2	1.09 (0.92,1.30)	P=0.304	10.4%	0.347	
Cohort	2	1.09 (0.92,1.30)	P=0.304	10.4%	0.347	
D-R	2	1.03 (0.90,1.18)	P=0.688		0.170	
Lymphoid	2	1.11 (0.90,1.37)	P=0.314	32.7%	0.216	
Leukemia	1	1.06 (0.56,2.00)	P=0.858			
**Glioma**	2	**0.81 (0.66,0.99)**	**P=0.041**	0%	0.774	
Cohort	2	**0.81 (0.66,0.99)**	**P=0.041**	0%	0.774	
D-R	2	0.93 (0.79,1.08)	P=0.325		0.750	

SSB: sugar-sweetened beverages; CC: case-control; D-R: dose-response analysis; EC: esophageal cancer; GC: gastric cancer; ESCC: esophageal squamous cell carcinoma; EADC: esophageal adenocarcinoma; IHBC: intrahepatic bile duct; GBTC: biliary track cancer; EHBC: extrahepatic bile duct; HCC: hepatocellular carcinoma; CRC: colorectal cancer.

**Table 2:** Fruit juice consumption and cancer risk

Factors	Number of studies	Random-effect	P value	Heterogeneity		Begg's/Egger's
		Pooled RR (95%CI)		I^2^	p	
**Fruit juice**						
***Total***	17	1.05 (0.95,1.16)	0.338	68.4%	0.000	0.650/0.442
Cohort	11	**1.06 (1.01,1.11)**	**0.013**	7.2%	0.376	0.029/0.018
CC	6	0.76 (0.48,1.20)	0.240	86.6%	0.000	
D-R	7	**1.14 (1.06,1.23)**	**0.000**		**0.447**	
**CRC**						
	4	0.87 (0.42,1.81)	0.715	90.3%	0.000	
CC	2	0.51 (0.07,3.77)	0.511	96.6%	0.000	
Cohort	2	1.32 (0.92,1.88)	0.123	0%	0.392	
D-R	2	**1.32 (1.01,1.74)**	**0.045**		**0.897**	
**Pancreatic**						
	3	0.96 (0.69,1.35)	0.362	63.3%	0.066	
CC	1	0.72 (0.53,0.97)	0.031			
Cohort	2	1.12 (0.88,1.42)	0.362	0%	0.545	
D-R	2	1.23 (0.83,1.93)	0.272		0.662	
**Breast**						
	3	1.06 (0.93,1.20)	0.375	0%	0.755	
Cohort	3	1.06 (0.93,1.20)	0.375	0%	0.755	
**Prostatic**						
	4	1.08 (0.95,1.22)	0.250	22.9%	0.274	
Cohort	4	1.08 (0.95,1.22)	0.250	22.9%	0.274	
D-R	3	1.38 (1.02,1.87)	0.036		0.360	
**Renal**						
	2	1.20 (0.93,1.54)	0.165	15.6%	0.277	
Cohort	2	1.20 (0.93,1.54)	0.165	15.6%	0.277	
D-R	2	1.15 (0.88,1.50)	0.302		0.334	

CC: case-control; D-R: dose-response analysis; CRC: colorectal cancer.

**Table 3:** SSB consumption and cancer mortality

Factors	Number of studies	Random-effect	P value	Heterogeneity		Begg's/Egger's
		Pooled RR (95%CI)		I^2^	p	
**Total**						
	11	**1.07 (1.01,1.14)**	**0.029**	61.8%	0.001	0.189/0.506
Cohort	10	**1.06 (1.00,1.12)**	**0.046**	50.9%	0.018	0.360/0.869
D-R	7	1.00 (0.98,1.03)	0.561		0.005	
**CRC**						
Cohort	6	1.09 (0.90,1.33)	0.715	64.7%	0.006	
D-R	2	1.01 (0.93,1.09)	0.867		0.024	
**Breast**						
Cohort	2	**1.17 (1.03,1.34)**	**0.017**	0%	0.611	
**Prostatic**						
Cohort	2	0.96 (0.79,1.17)	0.687	0%	0.791	
**Lung**						
Cohort	2	0.99 (0.86,1.13)	0.835	0%	0.557	

D-R: dose-response analysis; CRC: colorectal cancer.
