# Supplementary material for: Consumption of sugar-sweetened beverages and fruit juice and human cancer: a systematic review and dose-response meta-analysis of observational studies
Source: J Cancer. 2021 Mar 21;12(10):3077–88. doi: 10.7150/jca.51322 (PMC8040874; doi:10.7150/jca.51322)
Supplement: Supplementary file 1 — Supplementary figures and tables. [file jcav12p3077s1.pdf]

Supplementary table.

Table S1. General characteristics of included studies（n=71）

No.	Study	Study design	Location	Years / Follow-up	Exposure assessment	Gender/ Age	no. of study (case/control)	Type of cancer	Exposure	Covariate adjustments	NOS
**Sugar-sweetened beverages and cancer risk**											
1	Li 2017[16]	Case-Controls	USA	US:1993-1995<br>LA:1992-1997	FFQ	US:1001 men, 221 women/ 30-64 years<br>LA:1416 men, 418 women/30-74 years	500/2027	EC	Sweetened beverages OR 1.22(0.87, 1.70)	Adjusted for age, sex, race, study indicator, BMI, fruits and vegetables intake, cigarette smoking, GERD frequency and total energy intake.	5
							529/2027	GC	Sweetened beverages OR 1.21(0.86, 1.69)		
2	Yassibas 2012 [14]	Case-Controls	Turkey	March 2008 - June 2009	Questionnaires	132 men, 80 women/ cases:57.4±13.0years, controls: 57.9±12.5 years	106/106	GC	Colas OR 3.397(0.918, 12.568)<br>Other soft drinks OR 6.146(1.266, 29.832)	Adjusted for gender, residence, education, smoking, alcohol consumption and familial history of cancer or gastric cancer	5
3	Ren 2010 [66]	Cohort	USA	1995-1996; 8 years	Questionnaires	286,402 men, 195,161 women/ 50-71 years	392/481563	Oral	Carbonated soft drinks HR 0.77(0.54, 1.09)	Adjusted for age, sex, tobacco smoking, alcohol drinking, BMI, education, ethnicity, usual physical activity throughout the day, vigorous physical activity, and the daily intake of fruit, vegetables, red meat, white meat, and calories	8
							178/481563	Pharynx	Carbonated soft drinks HR 0.76(0.46, 1.25)		
							307/481563	Larynx	Carbonated soft drinks HR 0.82(0.55, 1.23)		
							438/481563	EC	Carbonated soft drinks ESCC:HR 0.85(0.46, 1.56); EADC: HR 1.11(0.66, 1.85)		
							455/481563	GC	Carbonated soft drinks: Gastric cardia: HR 0.89(0.55, 1.45); Gastric non-cardia: HR 0.75(0.45, 1.24)		
4	Pourfarzi 2009 [64]	Case-Controls	Iran	June 2003 - April 2005	A structured questionnaire	532 men, 195 women/ cases:65.4±11.5 years, controls: NA	217/394	GC	Fruit juice OR 1.29(0.73, 2.29)	Adjusted for gender, age group, education, family history of GC, citrus fruits, garlic, onion, red meat, fish, dairy products, strength and warmth of tea, preference for salt intake and H. pylori.	6

Table S1. *(Continued)*

No.	Study	Study design	Location	Years / Follow-up	Exposure assessment	Gender/ Age	no. of study (case/control)	Type of cancer	Exposure	Covariate adjustments	NOS
5	Ibiebele 2008 [15]	Case-Controls	Australia	2001-2005	FFQ	1668 men, 673 women/ 18-79 years	1102/1580	EC	Soft drinks EADC: OR 0.94(0.53, 1.66); ESCC: OR 0.40 (0.20, 0.78)	Adjusted for age; gender; body mass index; heartburn and acid reflux symptoms; cumulative history of smoking in pack years; alcohol intake status; educational status; total energy intake in kilojoules; and total vegetable intake	5
6	Mayne 2006 [55]	Case-Controls	USA	NA	In-person structured questionnaire	1411 men, 371 women/ cases:65-79 years, controls:30-64 years	488/687<br>607/687	EC<br>GC	Carbonated soft drinks EADC: OR 0.47(0.29, 0.76)<br>ESCC: OR 0.85 (0.48, 1.52)<br>Carbonated soft drinks Gastric cardia: HR 0.74(0.46, 1.16)<br>Gastric non-cardia: HR 0.65(0.43, 0.98)	Adjusted for age; sex; center; race; proxy interview status; average adult body mass index; mean caloric intake; consumption of beer, wine, and liquor; consumption of meat; cigarettes per day; education; income; and frequency of reflux symptoms.	5
7	Chazelas 2019 [29]	Cohort	France	2009-2017 / 5.1years (median)	Questionnaires	21533 men, 79724 women/ 18-72.7 years	1386/19931<br>332/25314<br>582/5383	Breast<br>CRC<br>Prostate	Sugary drinks HR 1.37(1.08, 1.73);<br>Artificially sweetened beverages HR 1.33(0.98, 1.75);<br>Fruit juice HR 1.13(0.91, 1.39)<br>Sugary drinks HR 1.07(0.63, 1.80);<br>Artificially sweetened beverages HR 0.80(0.44, 1.46);<br>Fruit juice HR 1.19(0.78, 1.82)<br>Sugary drinks HR 1.39(0.96, 2.02);<br>Artificially sweetened beverages HR 1.33(1.01, 1.75);<br>Fruit juice HR 1. 04(0.76, 1.42)	Adjusted for age, sex, energy intake without alcohol, sugar intake from other dietary sources, alcohol, sodium, lipid and fruit and vegetable intakes, body mass index, height, physical activity, smoking status, number of 24 hour dietary records, family history of cancer, educational level, and the following prevalent conditions at baseline, type 2 diabetes, hypertension, major cardiovascular event, the number of biological children, menopausal status at baseline, hormonal treatment for menopause at baseline and during follow-up, and oral contraception use at baseline and during follow-up.	8

Table S1. *(Continued)*

No.	Study	Study design	Location	Years / Follow-up	Exposure assessment	Gender/ Age	no. of study (case/control)	Type of cancer	Exposure	Covariate adjustments	NOS
8	Hodge 2018 [38]	Cohort	Australia	2003-2007 / 11.6 years (median)	FFQs	14101 men, 21485 women / 40-69 years	433/35593	Prostate	Sugary drinks HR 1.08(0.78, 1.50) Artificially sweetened beverages HR 0.81(0.49, 1.33)	Adjusted for country of birth, alcohol intake, smoking status, physical activity, Mediterranean diet score, and waist circumference.	9
							130/35593	Ovary	Sugary drinks HR 1.35(0.71, 2.56) Artificially sweetened beverages HR 1.37(0.72, 2.61)		
							146/35593	Kidney	Sugary drinks HR 1.48(0.87, 2.53) Artificially sweetened beverages HR 0.92(0.46, 1.84)		
							1055/35593	CRC	Sugary drinks HR 1.28(1.04, 1.57) Artificially sweetened beverages HR 0.79(0.60, 1.06)		
							946/35593	Breast	Sugary drinks HR 1.11(0.85, 1.45) Artificially sweetened beverages HR 0.95(0.73, 1.25)		
							167/35593	Endometrium	Sugary drinks HR 1.02(0.54, 1.91) Artificially sweetened beverages HR 0.81(0.42, 1.55)		
							165/35593	GC	Sugary drinks HR 1.17(0.73, 1.89) Artificially sweetened beverages HR 1.03(0.53, 1.98)		
9	Makarem 2018 [53]	Cohort	USA	1991-1995 / until 2013	FFQ	1222 men, 1962 women/ 26-84 years	248/3378	Breast	Sugary drinks HR 1.00(0.65, 1.57); Fruit juice HR 1.03(0.67, 1.62)	Adjusted for age, sex, smoking, alcohol, energy, fiber intake and red and processed meat intake. Additional adjustment for BMI, waist circumference, chronic diseases (CVD and diabetes), education, physical activity, antioxidant use.	7
							314/2990	prostate	Sugary drinks HR 1.36(0.88, 2.09); Fruit juice HR 1.58(1.04, 2.41)		
							133/6368	CRC	Sugary drinks HR 1.39(0.68, 2.82); Fruit juice HR 1.66(0.88, 3.12)		

Table S1. *(Continued)*

No.	Study	Study design	Location	Years / Follow-up	Exposure assessment	Gender/ Age	no. of study (case/control)	Type of cancer	Exposure	Covariate adjustments	NOS
10	Romanos 2018 [68]	Cohort	Spain	2014-2016/ 10.0 years (average)	FFQ	10713 women/ median age 33 years	100/10613	Breast	Sugar-sweetened beverages HR 1.41(0.77, 2.57; p=0.245)	Adjusted for eight, number of relatives with history of BC, smoking status, physical activity, alcohol intake, BMI, age of menarche, menopause, number of pregnancies of more than 6 months, pregnancy before the age of 30 years, months of breastfeeding, use of hormone replacement therapy and its duration and years at university.	9
11	Marzbani 2019 [54]	Case-Co ntrols	Iran	2013-2015	Questionnaire	620 women/ under 50 years	424/816	Breast	Soft drinks OR 2.8(1.9, 4.3); Industrially produced juices OR 2.7(1.1, 6.5)	Adjusted for demographic variables including age, gender, education level, and body mass index	6
12	Farvid 2016 [34]	Cohort	USA	1991-1992/ until 2013	FFQ	44233 women/ 27-44 years	3235/44223	Breast	Fruit juice HR 1.02(0.86, 1.21; P=0.85)	smoking, race, parity and age at first birth, height, BMI at age 18, weight change since age 18, age at menarche, family history of breast cancer, history of benign breast disease, oral contraceptive use, adolescent alcohol intake, adult alcohol intake, adolescent energy intake.	9
13	Chandran 2014 [28]	Case-Co ntrols	USA	NY:2002-20 08 NJ:2006-201 2	FFQ	3148 women/ 20-75 years	1558/3148	Breast	Sugary drinks African American OR 0.97(0.74, 1.27; P=0.45); European American OR 1.31(0.91, 1.89; P=0.15)	Adjusted for age, ethnicity, country of origin, education, age at menarche, menopausal status, parity, age at first birth, breastfeeding status, family history of breast cancer, HRT use, OC use, history of benign breast disease, study site, BMI, total MET hours per week, total energy intake.	7
14	Potischman2 002 [63]	Case-Co ntrols	USA	1990-1992	FFQ	2019 women/ 20-44 years	568/1451	Breast	Sugar-drinks 1.09(0.8, 1.5)	Adjusted for age at diagnosis, study site, race, education, alcohol consumption, years of oral contraceptive use, smoking status, BMI, and energy.	5

Table S1. *(Continued)*

No.	Study	Study design	Location	Years / Follow-up	Exposure assessment	Gender/ Age	no. of study (case/control)	Type of cancer	Exposure	Covariate adjustments	NOS
15	Stepien 2016 [74]	Cohort	European	1992-1998/ 11.4 years (average)	Questionnaires	142,194 men, 335,012 women/ NA	191/476713	HCC	Soft drinks HR 1.83(1.11, 3.02; P=0.01); Juice HR 1.38(0.80, 2.38; P=0.02)	Adjusted for non-alcoholic energy intake and stratified by age, sex and study centre, BMI, sex-specific physical activity, education level, alcohol at recruitment and alcohol intake pattern, smoking intensity, duration and history, diabetes status.	8
							302/476713	BTC	IHBC: Soft drinks HR 0.97(0.90, 1.06); Juice HR 1.04(1.00, 1.08) GBTC: Soft drinks HR 0.96(0.91, 1.00); Juice HR 0.99(0.95, 1.03)		
16	Larsson 2016 [49]	Cohort	Sweden	1987-1990/13.4 years (average)	Questionnaire	39,574 men, 31,258 women/ 45-83 years	148/70832	BTC	IHBC: sweetened beverages HR 1.69(0.41, 7.03; P=0.37); EHBC: sweetened beverages HR 1.79(1.02, 3.13; P=0.05); Gallbladder: sweetened beverages HR 2.24(1.02, 4.89; P=0.02)	Adjusted for age, sex education, smoking, body mass index, dietary protein intake, and total energy intake	8
17	Pacheco 2019 [61]	Cohort	USA	1995-1996/ 20.1 years (median)	FFQ	99798 women/ 52.0±13.5 years	1318/99798	CRC	Sugar-sweetened beverage HR 1.16(0.87, 1.54)	Adjusted for age, race/ethnicity, socioeconomic status, total years smoked, alcohol intake, colorectum cancer family history of first-degree relatives, history of polyps, diabetes, physical activity, aspirin use, multivitamin use, menopausal status, menopausal hormone therapy use, oral contraceptive use, body mass index, total energy intake, and dietary variables: red meat, processed meat, and vegetable intakes.	8
18	Mahfouz 2014 [52]	Case-Controls	Egypt	2010-2011	FFQ	216 men, 234 women/ NA	150/300	CRC	Artificial sweeteners OR 20.8(2.7, 159.7; P=0.003); Soft drinks OR 4.6(1.9, 11.01; P=0.001); fruit juice OR 0.18(0.09, 0.36)	NA	6

Table S1. *(Continued)*

Study	Study	Study design	Location	Years / Follow-up	Exposure assessment	Gender/ Age	no. of study (case/control)	Type of cancer	Exposure	Covariate adjustments	NOS
19	Theodoratou2014 [76]	Case-Controls	UK	1999-2006	FFQ	NA/ 16-79 years	2062/2776	CRC	Sugar-sweetened beverages OR 1.12(1.05, 1.19; P=0.0003)	NA	7
20	Annema 2011 [22]	Case-Controls	Australia	2005-2007	FFQ	1065 men, 708 women/ 40-79 years	834/939	CRC	Fruit juice OR 1.38(1.08, 1.75; P=0.015)	Adjusted for sex, age, body mass index at age 20 y, energy intake, multivitamin use, alcohol consumption, physical activity, smoking, diabetes, and socioeconomic status.	6
21	Zhang 2010 [78]	Cohort	USA	1976-2003/ up to 6-20 years	FFQ	239,193 men, 492,248 women/ 25-93 years	4895/731,441	Colon	Sugar-sweetened carbonated soft drinks OR 0.94(0.66, 1.32)	Adjusted for education; smoking habits; height; body mass index; physical activity; family history of colorectal cancer; use of nonsteroidal anti-inflammatory drugs; multivitamin use; red meat intake; total milk consumption; alcohol consumption; dietary folate intake; total energy intake; and in women, oral contraceptive use; and postmenopausal hormone use.	7
22	Fung 2010 [35]	Cohort	USA	NHS:1980-2006; HPFS:1986-2006/ up to 20-26 years	FFQs	45,490 men, 87,256 women/ 30-75 years	2464/132,746	CRC	Sweetened beverage<br>Men: OR 1.17(1.02, 1.33)<br>Women: OR 1.04(0.94, 1.16)	NA	8
23	Bener 2010 [24]	Case-Controls	UK	2008-2009	Questionnaire	249 men, 179 women/ 18-82 years	146/282	CRC	Soft drinks OR 1.62(1.19, 2.17; P=0.02)	NA	7
24	Rizk 2019 [68]	Case-Controls	France	2008-2012	Questionnaire	423 men, 159 women/ 51.8-70.1 years	181/401	HCC	Carbonated beverages OR 2.44(1.17, 5.09; P=0.021)	Adjusted for age, gender, center, total energy from nonalcoholic sources, cirrhosis diagnosis, Child-Pugh score, diabetes, etiology of cirrhosis and occupational physical activity.	6

Table S1. *(Continued)*

No.	Study	Study design	Location	Years / Follow-up	Exposure assessment	Gender/ Age	no. of study (case/control)	Type of cancer	Exposure	Covariate adjustments	NOS
25	Polesel 2013 [62]	Case-Controls	Italy	1992-2008	A structured questionnaire	628 men, 164 women/18-76 years	198/594	Nasopharyngeal	Soft drinks OR 0.93(0.60, 1.45; P=0.70);	Adjusted for center, sex, age, place of living, year of interview, education, tobacco smoking, alcohol drinking, and non-alcohol energy	7
26	Sanchez 2003 [70]	Case-Controls	Spain	1996-1999	A structured questionnaire	608 men, 142 women / 20-91 years	375/375	Oropharynx cancer	Fruit juice OR 0.72(0.47, 1.08; P=0.18);	NA	6
27	Mccullough 2014 [56]	Cohort	USA	1999-2009/ 10 years	FFQ	43,350 men, 57,092 women/47-95 years	1196/100442	Lymphoma	Sugar-sweetened carbonated OR 1.10(0.77, 1.58; P=0.62); Artificial sweetened carbonated beverage OR 0.92(0.73, 1.17; P=0.14)	Adjusted for age, gender, history of diabetes, BMI, smoking status, energy intake, and artificially sweetened carbonated beverage intake.	7
28	Schernhammer 2012 [71]	Cohort	USA	NHS:1984-2006 HPFS:1986-2006	FFQ	51,529 men, 121,701 women/ 30-75 years	1948/125028	Lymphoma and leukemia	Sugar-sweetened soda NHL: OR 1.34(0.98, 1.83; P=0.05); Multiple myeloma: OR 1.47(0.76, 2.83; P=0.31); leukemia: OR 1.06(0.56, 2.00; P=0.68)	Adjusted for age; questionnaire cycle; diet soda consumption; fruit and vegetable consumption; multivitamin use; intakes of alcohol, saturated fat, animal protein, and total energy; race; BMI; height; discretionary physical activity; smoking history; and menopausal status and use of hormone replacement therapy (women only).	8
29	Inoue-choi 2013 [41]	Cohort	USA	1986-2010	FFQ	23,039 women/ 52-71 years	595/23039	Endometrial	Sugar-sweetened beverage and fruit juice: type I HR 1.54(1.12, 2.12; P=0.008).	Adjusted for age, smoking, physical activity, alcohol use, estrogen use, age at menarche, age at menopause, number of live births, history of diabetes, and coffee intake, BMI.	7

Table S1. *(Continued)*

No.	Study	Study design	Location	Years / Follow-up	Exposure assessment	Gender/ Age	no. of study (case/control)	Type of cancer	Exposure	Covariate adjustments	NOS
30	King 2013 [45]	Case-controls	USA	2002-2006	FFQ	822 women/ 55+years and 65+years	417/295	Endometrial	Sugary drinks OR 1.48(0.94, 2.33; P=0.14)	Adjusted for age, energy intake, education, race, age at menarche, menopausal status and age at menopause for postmenopausal women, parity, oral contraceptive use, HRT use, BMI, smoking status and pack-years for ever smokers, physical activity.	7
31	King 2013 [46]	Case-controls	USA	2001-2008	FFQ	595 women/ cases:21+ years, controls:55+years and 65+years	205/390	Ovarian	Sugary drinks OR 1.09(0.65, 1.84; P=0.47)	Adjusted for age, energy intake, education, race, age at menarche, menopausal status and age at menopause for postmenopausal women, parity, oral contraceptive use, HRT use, BMI, smoking status, tubal ligation, and first degree relative with ovarian cancer.	6
32	Navarrete 2016 [59]	Cohort	European	1992-2000; 11.6 years (median)	Questionnaires	142,202 men, 334,997 women/ mean age 51 years	865/477199	Pancreatic	Total sweet beverage OR 0.92(0.72, 1.17; P=0.17)	Adjusted for educational level, physical activity, smoking status, and alcohol consumption (g/d), diabetes, energy intake, and BMI	7
33	Gallus 2010 [36]	Case-controls	Italy	1991-2008	FFQ	522 men, 455 women/ median age 63 years	325/652	Pancreatic	Carbonated drink OR 1.02(0.72, 1.44)	Adjusted for sex and age, year of interview, education, body mass index, tobacco smoking, alcohol drinking, total energy intake, family history of pancreatic cancer, and history of diabetes.	7
34	Mueller 2010 [58]	Cohort	Singapore	1999-2004/ 10.7 years (average)	A structured questionnaire	NA	140/648387	Pancreatic	Soft drinks HR 1.87(1.10, 3.15; P=0.02); Juice HR 1.31(0.74, 2.30); P=0.35);	Adjusted for age, sex, ethnicity, and year of interview, education, smoking index, moderate physical activity, alcohol, added sugar and candy, and total calories, type 2 diabetes mellitus and BMI.	9
35	Chan 2009 [27]	Case-control	USA	1995-1999	FFQ	1174 men, 1059 women/ 21-85 years	532/1701	Pancreatic	Sweetened beverage OR 1.0(0.7, 1.3; P=0.7)	Adjusted for age, sex, and energy intake, body mass index, race, education, smoking, history of diabetes, and physical activity, total red meat, white meat, vegetable and fruit, eggs, fish, dairy, whole grain, and refined grain.	6

Table S1. *(Continued)*

No.	Study	Study design	Location	Years / Follow-up	Exposure assessment	Gender/ Age	no. of study (case/control)	Type of cancer	Exposure	Covariate adjustments	NOS
36	Bao 2008 [23]	Cohort	USA	1995-1996/ 7.2 years (average)	FFQ	284,076 men, 203,846 women/ 50-71 years	1258/487922	Pancreatic	Sugar-sweetened beverage OR 0.83(0.67,1.04; P=0.10).	Adjusted for sex, race, education, BMI, alcohol, smoking, physical activity, energy-adjusted red meat consumption, energy-adjusted folate intake, and total energy	8
37	Nothling 2007 [60]	Cohort	Germany	1993-1996/ 8 years	FFQ	72,813 men, 89,337 women/ 45-75 years	434/161716	Pancreatic	Fruit juice OR 1.08(0.83,1.41; P=0.56).	Adjusted for race-ethnicity, age at cohort entry, smoking status, pack-years of smoking, family history of pancreatic cancer, energy intake, intakes of red meat and processed meat, and BMI.	8
38	Larsson 2006 [48]	Cohort	Sweden	1997-2005/ 7.2 years (average)	FFQ	42,524 men, 35,273 women/45-83 years	131/77797	Pancreatic	Soft drink HR 1.93(1.18,3.14; P=0.02);	Adjusted for age, sex, education, smoking status and pack-years, BMI, and intakes of total energy and alcohol.	8
39	Schernhammer 2005 [72]	Cohort	USA	NHS:1980-2000; HPFS:1986-2000/up to 20years	FFQ	49,364 men, 88,794 women/ 30-75 years	379/138158	Pancreatic	Soft drink HR 1.13(0.81,1.58; P=0.47);	Adjusted for age in years, BMI, gender, follow-up cycle, history of diabetes, smoking status in nine categories, quintiles of caloric intake, quintiles of physical activity, and other soft drink consumption.	7
40	Chan 2005 [26]	Case-control	USA	1995-1999	FFQ	1171 men,1056 women/ 21-85 years	526/1701	Pancreatic	Fruit juice OR 0.72(0.54,0.98; P=0.05);	Adjusted for age, sex and energy intake.	6
41	Lyon 1992 [51]	Case-control	USA	1984-1987	A standardized questionnaire	NA/ 40-79 years	149/363	Pancreatic	Caffeine-containing soft drinks OR 1.31(0.89, 1.94)	NA	5
42	Turati 2015 [27]	Case-control	Italy	2003-2014	A structured questionnaire	1156 men, 199 women/ 25-80 years	690/665	Bladder	Cola drinks OR 1.04(0.73, 1.49)	Adjusted for age, sex, study center, year of interview, smoking, education, alcohol drinking, body mass index, family history	7
43	Lee 2007 [50]	Cohort	USA	1980-1986/ up to 7-20 years	FFQ	244,483 men, 530,469 women/ NA	1478/774952	Renal	Soda drinks OR 1.11(0.89, 1.38; P=0.24)	Adjusted for age, history of hypertension, body mass index, pack-years of smoking, combination of parity and age at first birth, fruit and vegetable consumption, alcohol intake and total energy intake.	7

Table S1. *(Continued)*

No.	Study	Study design	Location	Years / Follow-up	Exposure assessment	Gender/ Age	no. of study (case/control)	Type of cancer	Exposure	Covariate adjustments	NOS
44	Rashidkhani2004 [65]	cohort	Sweden	1987-1990/ 13.4 years (average)	FFQ	61,000 women/ 40-76 years	122/61,000	Renal	Fruit juice RR 1.46(0.94, 2.26; P=0.26)	Adjusted for age and body mass index	8
45	Miles 2018 [57]	Cohort	USA	1993-2001/ 9 years (median)	Baseline (BQ) and diet history (DHQ) questionnaires	22,720 men/ 55-74 years	1996/22720	Prostate	Sugar-sweetened beverage HR 1.21(1.06, 1.39); Fruit juice HR 1.07(0.94, 1.22).	Adjusted for age, race, study center, BMI, education, smoking, family history of prostate cancer, history of diabetes, PSA screening, and energy intake	8
46	Darke 2012 [31]	Cohort	Sweden	1992-2009/ 14.9 years (median)	Questionnaires	8128 men/ 45-73 years	817/8128	Prostate	Fruit juice OR 0.99(0.81, 1.22); Sugar-sweetened beverages OR 1.13(0.92, 1.38)	Adjusted for age, year of study entry, season of data collection, energy intake, height, waist, physical activity, smoking, educational level, birth in Sweden, alcohol, calcium, and selenium.	7
47	Sharpe 2002 [73]	Case-control	Canada	1979-1985	Questionnaires	252 men/ 40-70 years	115/137	Prostate	Carbonated beverages OR 1.0(0.7, 1.4)	Adjusted for age, ethnicity, respondent status, family income, body mass index, cumulative cigarette smoking, and cumulative alcohol consumption.	6
48	Ellison 2000 [33]	Cohort	Canada	1970-1972/ up to 1993	Dietary interviews	3400 men/ 50-84 years	145/3400	Prostate	Cola OR 1.29(0.74, 2.26)	Adjusted for five-year age group	7
49	Jain 1998 [42]	Case-control	Canada	1989-1993	A structured questionnaire	1253 men/ cases:69.8±7.3 years, controls: 69.9±7.3years	617/636	Prostate	Cola OR 0.79(0.53, 1.17)	Adjusted for age and total energy intake	6
50	Genkinger 2012 [37]	Cohort	European	NA/ 3-16years	FFQ	318875men, 537204women/ 15-107 years	2057/NA	Pancreatic	Sugar-sweetened carbonated soft OR 1.19(0.98, 1.46)	Adjusted for r smoking status, alcohol intake, history of diabetes, BMI, and energy intake, age and years and year of questionnaire return	8

Table S1. *(Continued)*

No.	Study	Study design	Location	Years / Follow-up	Exposure assessment	Gender/ Age	no. of study (case/control)	Type of cancer	Exposure	Covariate adjustments	NOS
51	Lagergren 2006 [47]	Case-control	Swedish	1995-1997	FFQ	Men and women/ any	177/784	EC	Carbonated drinks OR 1.15(0.67, 2.00)	Adjusted for reflux symptoms, body mass index , tobacco smoking status, alcohol use, socioeconomic status, and dietary intake of fruits and vegetables.	6
							255/784	GC	Carbonated drinks OR 1.10(0.70, 1.72)		
52	Hu 2009 [40]	Case-control	Canada	1994-1997	Questionnaires	3164men,3013 women/ ⩾20 years	1117/4897	Renal	Soft drinks OR 1.26(0.96, 1.67)	Adjusted for 10-year age groups, province, education, body mass index, sex, pack-years of smoking, alcohol drinking (g/day), total consumption of meat, total consumption of vegetables and fruit, and total energy intake.	6
53	Talamini 1990 [75]	Case-control	Italy	1986-1989	Questionnaires	595men, 310women/ 20-74 years	5/20	Renal	Colas OR 0.72(0.27, 1.97)	Adjusted for age, sex, education, area of residence, and BMI	5
54	Ros 2011 [26]	Cohort	European	1992-2000/ 9.3years(mean)	Dietary questionnaire	68276men,165637women/ 25-70 years	494/233236	Bladder	Soft drinks OR 1.03(0.83, 1.30)	Adjusted for energy intake, smoking status, duration of smoking and lifetime intensity of smoking.	8
55	Jensen 1986 [43]	Case-control	Denmark	1979-1981	A structured questionnaire	857men,285women/ any	371/771	Bladder	Soft drinks OR males 1.8(0.9, 3.5); females 0.7(0.1, 3.8)	Adjusted for smoking and age	6
56	Jiang 2008 [44]	Case-control	USA	1987-1999	A structured questionnaire	616men,233women/ 25-64 years	967/970	Bladder	Soda OR 1.39(0.87, 2.21)	Adjusted for level of education, use of NSAIDs, intake of carotenoids, number of years as a hairdresser/barber, cigarette smoking status, duration of smoking, and intensity of smoking.	5
57	Stefani 2007 [30]	Case-control	Uruguay	1996-2000	FFQ	666men,90women/ 30-89 years	255/501	Bladder	Soft OR 1.1(0.7, 1.7)	Adjusted for age, sex, residence, urban/rural status, education, family history of bladder cancer among first-degree relatives, BMI, occupation, smoking status, years since quitting, number of cigarettes smoked per day, *maté* drinking, milk intake, and, when appropriate, coffee drinking and tea drinking	6

Table S1. *(Continued)*

No.	Study	Study design	Location	Years / Follow-up	Exposure assessment	Gender/ Age	no. of study (case/control)	Type of cancer	Exposure	Covariate adjustments	NOS
58	Bruemmer 2013 [25]	Case-control	UK	1987-1990	A structured questionnaire	422men, 245women/ 45-65years	262/405	Bladder	Soft drinks OR: males 1.6(0.7, 3.6); females 2.3(0.8, 6.3)	Adjusted for r age, county, and smoking	7
59	Holick 2010 [39]	Cohort	USA	1980-2005/ 24 years	FFQ	36625men, 147353women/ 40-75years	335/610832	Glioma	Carbonated beverages OR: caffeinated 0.75(0.51, 1.11); decaffeinated 0.75(0.49, 1.17)	Adjusted for age and total caloric intake.	8
60	Dubrow 2012 [32]	Cohort	USA	1995-1996/ up to December 2006	Questionnaires	Men and women/ 50-71years	343/545771	Glioma	Soda OR 0.87(0.65, 1.15)	Adjusted for age, sex, race/ethnicity, energy intake, height, fruit and vegetable intake, and nitrite intake from plant sources	8
**Sugar-sweetened beverages and cancer mortality**											
61	Zhang 2020 [89]	Cohort	China	1999-2014/ 7.9 years	Questionnaire	Men and women/ ≥20years	1765/66026	Overall cancer	Sugar-sweetened beverages HR 1.05(0.80,1.39)	Adjusted for age, sex, family income-poverty ratio level, race, education level, marital status, alcohol consumption, smoking, and leisure-time physical activity, body mass index, prevalent high cholesterol level, hypertension, and diabetes, and history of cardiovascular disease and cancer.	8
62	Tasevska 2014 [88]	Cohort	USA	1995-1996/ 13 years	Questionnaire	Men and women/ 50-71years	17730/353751	Overall cancer	Sugar-sweetened beverages Women: HR 1.04(0.96,1.13); Men: HR 1.00(0.94,1.07)	Adjusted for age, BMI, marital status, smoking, race, education, physical activity, and intake of energy, vegetables, alcohol, family history of cancer and intakes of total fat and red meat.	8
63	Odegaard 2015 [87]	Cohort	China	1993-1998/ Follow up to 2011	Questionnaires	Men and women/ 45-74 years	4092/52584	Overall cancer	Soft drink HR 0.85(0.69,1.05)	Adjusted for age, sex, dialect, education, year of interview, smoking, moderate and vigorous activity, sleep, BMI, hypertension, non-beverage vegetable-fruit-soy–rich dietary pattern score, and energy intake.	7

Table S1. *(Continued)*

No.	Study	Study design	Location	Years / Follow-up	Exposure assessment	Gender/ Age	no. of study (case/control)	Type of cancer	Exposure	Covariate adjustments	NOS
64	Mullee 2019 [86]	Cohort	European	1992-2000/ 16.4 years	Questionnaires	Men and women/ 50.8 years	18003/451743	Overall cancer	Soft drink HR 1.02(0.95,1.11)	Adjusted for body mass index, calculated as weight in kilograms divided by height in meters squared, physical activity index, educational status, alcohol consumption, smoking status and intensity, smoking duration, ever use of contraceptive pill, menopausal status, ever use of menopausal hormone therapy, intakes of total energy, red and processed meat, fruits and vegetables, coffee, and fruit and vegetable juice, stratified by age, EPIC center, and sex.	9
65	Malik 2019 [84]	Cohort	USA	From 1980/ Follow up to 2014	FFQ	Men and women/ 40-75 and 30-55 years	12380/118363	Overall cancer	Sugar-sweetened beverages HR 1.16(1.04,1.29); Artificially sweetened beverages HR 1.04(0.96,1.12);	Adjusted for: age, smoking, alcohol intake, postmenopausal hormone use (NHS), physical activity, family history of diabetes, family history of myocardial infarction, family history of cancer, multivitamin use, ethnicity, aspirin use, baseline history of hypertension and hypercholesterolemia; intake of whole grains, fruit, vegetables, red and processed meat; total energy; and body mass index.	8
66	Khan 2004 [83]	Cohort	Japan	1984-2002/ NA	Questionnaires	Men and women/ ⩾40 years	1010/3158	Overall cancer	Carbonated drink RR Men 0.9 (0.6, 1.3); Women 1.5 (0.9, 2.3);	NA	5
67	Guercio 2018 [82]	Cohort	USA	1999-2001/ 7.3 years	FFQ	Men and women/ ≥21 years	392/1264	CRC	Artificially sweetened beverages HR 0.58(0.31, 1.07);	Adjusted for age, depth of invasion through bowel wall, number of positive lymph nodes, baseline performance status, chemotherapy treatment group, consistent aspirin use, and time-varying physical activity, and body mass index, time-varying total calorie intake.	6

Table S1. *(Continued)*

No.	Study	Study design	Location	Years / Follow-up	Exposure assessment	Gender/ Age	no. of study (case/control)	Type of cancer	Exposure	Covariate adjustments	NOS
68	Fung 2014 [81]	Cohort	USA	From 1986-2010	FFQ	Women 30-55 years	NA/121701	CRC	Sugary drinks HR 1.11 (1.01, 1.23)	Adjusted for age, physical activity, BMI, weight change, cancer grade, chemotherapy, smoking status, energy intake, colon or rectal cancer, stage of disease, date of colorectal cancer diagnosis, an AHEI-2010 score without the specific component.	6
69	Fuchs 2014 [80]	Cohort	USA	1999-2001/ 7.3 years	FFQ	Men and women/ ≥21 years	262/1011	CRC	Sugar-sweetened beverages HR 1.67(1.04,2.68)	Adjusting with Cox proportional hazards regression for age, sex, depth of invasion through bowel wall, number of positive lymph nodes, baseline performance status, treatment group, and the following time-varying covariates: total energy intake, body mass index, physical activity level, Western dietary pattern, and prudent dietary pattern.	6
70	Barrington 2016 [79]	Cohort	USA	2000-2002/ 6.9 years	FFQ	Men and women/ 50-76	1933/69582	Overall cancer	Sugar-sweetened beverages HR 1.23(1.04,1.47)	Adjusted for age, sex, race/ethnicity, marital status, education, annual income, BMI at age 45 years, current use of cholesterol-lowering medication, aspirin use in last 10 years, non-aspirin non-steroidal anti-inflammatory drug use in last 10 years, average physical activity in 10 years before baseline, smoking status, average alcohol intake, mammogram in past 2 years, prostrate-specific antigen test in the last 2 years, sigmoidoscopy in the last 10 years, number of servings/d of fruits, number of servings/d of vegetables and total daily energy intake, history of cancer other than non-melanoma skin cancer, family history of cancer, years of oestrogen therapy, years of oestrogen plus progestin therapy, age at menopause and age at menarche.	7

Table S1. *(Continued)*

No.	Study	Study design	Location	Years / Follow-up	Exposure assessment	Gender/ Age	no. of study (case/control)	Type of cancer	Exposure	Covariate adjustments	NOS
71	Miles 2016 [85]	Case-control	USA	1999-2004/ 12.1 years	Questionnaires	Men and women/ 18-65	240/1641	UADT	Sugary drinks<br>HR 1.97(1.32,2.93)	Adjusted for age, gender, ethnicity, education, smoking, alcohol drinking, caloric intake, pathology type, and tumor differentiation grade.	6

EC: esophageal cancer; GC: gastric cancer; FFQ: food frequency questionnaires; ESCC: esophageal squamous cell carcinoma; EADC: esophageal adenocarcinoma; IHBC: intrahepatic bile duct; GBTC: biliary tract cancer; EHBC: extrahepatic bile duct; UADT: upper aerodigestive tract.

Table S2. Subgroup analysis of sugar-sweetened beverages consumption and cancer risk

Subgroup	No. of included studies	I^2	Random-effect model		
			OR	95%CI	P
All studies					
**Geographic location**					
European	19	58.2%	**1.16**	**(1.06,1.27)**	**0.001**
North America	28	59.0%	1.06	(0.98,1.15)	0.147
Oceania	2	33.6%	0.97	(0.66,1.45)	0.898
Asia	2	31.4%	2.39	(1.64,3.48)	**<0.001**
South America	1				
Africa	1				
**No. of cases**					
<500	28	69.5%	**1.25**	**(1.12,1.41)**	**<0.001**
⩾500	25	59.7%	1.06	(0.99,1.13)	0.076
**Type of FFQ**					
validated	27	41.8%	**1.10**	**(1.04,1.18)**	**0.002**
unvalidated	26	75.6%	**1.10**	**(1.04,1.30)**	**0.007**
**Study quality score**					
High (>6)	34	55.3%	**1.09**	**(1.03,1.15)**	**0.002**
Low (⩽6)	19	75.4%	**1.25**	**(1.03,1.52)**	**0.025**
Cohort studies					
**Geographic location**					
European	8	59.1%	1.10	(0.98,1.24)	0.091
North America	16	62.2%	1.06	(0.96,1.16)	0.246
Oceania	1				
Asia	1				
**No. of cases**					
<500	12	60.1%	1.10	(0.98,1.23)	0.123
⩾500	14	60.7%	1.08	(0.99,1.17)	0.075
**Type of FFQ**					
validated	15	56.3%	**1.10**	**(1.00,1.20)**	**0.043**
unvalidated	11	64.7%	1.06	(0.96,1.17)	0.239
**Study quality score**					
High (>6)	26	59.3%	**1.08**	**(1.01,1.15)**	**0.020**
Low (⩽6)	0				

Table S2. *(Continued)*

Subgroup	No. of included studies	I^2	Random-effect model		
			OR	95%CI	P
Case-control studies					
**Geographic location**					
European	12	74.6%	**1.40**	**(1.13,1.73)**	**0.002**
North America	12	57.7%	1.07	(0.92,1.24)	0.380
Asia	1				
Oceania	1				
South America	1				
Africa	1				
**No. of cases**					
<500	16	65.1%	**1.43**	**(1.16,1.76)**	**0.001**
⩾500	11	62.2%	1.03	(0.90,1.17)	0.673
**Type of FFQ**					
validated	12	7.4%	**1.12**	**(1.04,1.21)**	**0.003**
unvalidated	15	80.0%	1.31	(1.02,1.67)	0.032
**Study quality score**					
High (>6)	8	23.6%	1.15	(1.03,1.52)	0.015
Low (⩽6)	19	75.4%	**1.25**	**(1.03,1.29)**	**0.025**
EC					
**Geographic location**					
European	1				
North America	3	63.1%	0.87	(0.61,1.25)	0.445
Oceania	1				
**No. of cases**					
<500	1				
⩾500	4	62.8%	0.80	(0.58,1.11)	0.182
**Type of FFQ**					
validated	3	65.2%	0.90	(0.58,1.40)	0.649
unvalidated	2	51.4%	0.77	(0.52,1.14)	0.194
**Study quality score**					
High (>6)	1				
Low (⩽6)	4	69.3%	0.79	(0.54,1.16)	0.974

Table S2. *(Continued)*

Subgroup	No. of included studies	I^2	Random-effect model		
			OR	95%CI	P
GC					
**Geographic location**					
European	2	67.9%	2.34	(0.77,7.08)	0.132
North America	3	37.5%	0.85	(0.66,1.08)	0.190
Oceania	1				
**No. of cases**					
<500	2	67.9%	2.34	(0.77,7.08)	0.132
≥500	4	23.5%	0.90	(0.74,1.10)	0.323
**Type of FFQ**					
validated	3	0.0%	1.15	(0.92,1.44)	0.208
unvalidated	3	59.7%	0.93	(0.63,1.38)	0.721
**Study quality score**					
High (>6)	2	0.0%	0.94	(0.73,1.22)	0.664
Low (≤6)	4	63.4%	1.11	(0.78,1.56)	0.566
CRC					
**Geographic location**					
European	3	57.4%	1.17	(0.92,1.49)	0.196
North America	4	0.0%	**1.08**	**(1.00,1.17)**	**0.040**
Oceania	1				
Africa	1				
**No. of cases**					
<500	4	76.0%	1.56	(0.93,2.64)	0.095
≥500	5	42.4%	**1.10**	**(1.01,1.27)**	**0.022**
**Type of FFQ**					
validated	7	68.6%	1.16	(0.74,1.81)	0.070
unvalidated	2	63.2%	1.12	(0.99,1.26)	0.513
**Study quality score**					
High (>6)	8	44.5%	**1.11**	**(1.02,1.20)**	**0.017**
Low (≤6)	1				

Table S2. *(Continued)*

Subgroup	No. of included studies	I^2	Random-effect model		
			OR	95%CI	P
Pancreatic cancer					
**Geographic location**					
European	3	71.9%	1.16	(0.78,1.72)	0.468
North America	5	46.1%	1.06	(0.89,1.25)	0.515
Asia	1				
**No. of cases**					
<500	5	42.0%	**1.33**	**(1.05,1.69)**	**0.018**
≥500	4	51.6%	0.98	(0.82,1.17)	0.802
**Type of FFQ**					
validated	6	58.8%	1.24	(0.83,1.87)	0.370
unvalidated	3	69.8%	1.09	(0.90,1.32)	0.297
**Study quality score**					
High (>6)	7	66.2%	1.13	(0.92,1.38)	0.232
Low (≤6)	2	11.6%	1.11	(0.86,1.44)	0.414
Breast cancer					
**Geographic location**					
European	3	77.0%	**1.62**	**(1.11,2.37)**	**0.012**
North America	3	0.0%	1.03	(0.85,1.24)	0.385
Oceania	1				
**No. of cases**					
<500	4	73.2%	**1.50**	**(1.08,2.09)**	**0.014**
≥500	3	0.0%	1.06	(0.93,1.20)	0.410
**Type of FFQ**					
validated	5	0.0%	1.07	(0.94,1.21)	0.295
unvalidated	2	82.7%	**1.69**	**(1.08,2.64)**	**0.022**
**Study quality score**					
High (>6)	4	0.0%	1.11	(0.98,1.26)	0.089
Low (≤6)	3	81.7%	1.49	(0.99,2.23)	0.053

Table S2. *(Continued)*

Subgroup	No. of included studies	I^2	Random-effect model		
			OR	95%CI	P
Prostatic cancer					
**Geographic location**					
European	2	0.0%	**1.20**	**(1.03,1.39)**	**0.019**
North America	5	18.1%	1.10	(0.95,1.28)	0.206
Oceania	1				
**No. of cases**					
<500	4	0.0%	**1.18**	**(1.00,1.39)**	**0.047**
⩾500	4	32.2%	1.09	(0.94,1.25)	0.257
**Type of FFQ**					
validated	2	0.0%	1.01	(0.80,1.27)	0.932
unvalidated	6	0.0%	**1.16**	**(1.06,1.28)**	**0.001**
**Study quality score**					
High (>6)	6	0.0%	**1.17**	**(1.07,1.28)**	**0.001**
Low (⩽6)	2	0.0%	0.90	(0.70,1.17)	0.442
Renal cancer					
**Geographic location**					
European	1				
North America	2	0.0%	1.17	(0.98,1.38)	0.081
Oceania	1				
**No. of cases**					
<500	1				
⩾500	3	0.0%	**1.18**	**(1.00,1.38)**	**0.046**
**Type of FFQ**					
validated	2	0.0%	1.14	(0.94,1.38)	0.198
unvalidated	2	11.6%	1.18	(0.82,1.68)	
**Study quality score**					
High (>6)	2	0.0%	1.14	(0.94,1.38)	0.198
Low (⩽6)	2	0.0%	1.18	(0.82,1.68)	0.372

Table S2. *(Continued)*

Subgroup	No. of included studies	I^2	Random-effect model		
			OR	95%CI	P
Bladder cancer					
**Geographic location**					
European	4	7.9%	1.14	(0.93,1.39)	0.208
North America	1				
South America	1				
**No. of cases**					
<500	4	4.9%	1.16	(0.87,1.54)	0.169
⩾500	2	0.0%	1.15	(0.94,1.41)	0.311
**Type of FFQ**					
validated	1				
unvalidated	5	2.9%	1.15	(0.97,1,37)	0.101
**Study quality score**					
High (>6)	3	5.1%	1.09	(0.90,1.33)	0.391
Low (⩽6)	3	0.0%	1.30	(0.97,1.73)	0.075

EC: esophageal cancer; GC: gastric cancer; FFQ: food frequency questionnaires.

Table S3. Subgroup analysis of fruit juice consumption and cancer risk

Subgroup	No. of included studies	I^2	Random-effect model		
			OR	95%CI	P
**All studies**					
**Geographic location**					
European	6	47.2%	1.11	(0.96,1.28)	0.162
North America	7	16.7%	1.02	(0.96,1.09)	0.506
Asia	2	0%	1.30	(0.87,1.94)	0.201
Oceania	1				
Africa	1				
**No. of cases**					
<500	8	80.2%	0.99	(0.77,1.27)	0.926
⩾500	9	41.9%	1.07	(0.97,1.17)	0.173
**Type of FFQ**					
validated	9	79.6%	1.00	(0.85,1.18)	0.958
unvalidated	8	24.7%	1.09	(0.99,1.20)	0.094
**Study quality score**					
High (>6)	11	7.2%	**1.06**	**(1.01,1.11)**	**0.013**
Low (⩽6)	6	86.6%	0.76	(0.48,1.20)	0.240
**Cohort studies**					
**Geographic location**					
European	5	20.9%	**1.14**	**(1.02,1.28)**	**0.020**
North America	5	0%	1.03	(0.99,1.08)	0.174
Asia	1				
**No. of cases**					
<500	5	47.1%	1.10	(0.98,1.35)	0.079
⩾500	6	0%	**1.08**	**(1.01,1.16)**	**0.027**
**Type of FFQ**					
validated	6	0%	1.03	(0.99,1.08)	0.182
unvalidated	5	9.1%	**1.11**	**(1.02,1.21)**	**0.014**
**Study quality score**					
High (>6)	11	7.2%	**1.06**	**(1.01,1.11)**	**0.013**
Low (⩽6)	0				

Table S3. *(Continued)*

Subgroup	No. of included studies	I^2	Random-effect model		
			OR	95%CI	P
**Case-control studies**					
**Geographic location**					
European	1				
North America	2	0%	0.75	(0.57,0.98)	0.380
Oceania	1				
Africa	1				
Asia	1				
**No. of cases**					
<500	3	89.4%	0.56	(0.21,1.50)	0.253
⩾500	3	82.5%	0.97	(0.60,1.57)	0.898
**Type of FFQ**					
validated	3	94.2%	0.89	(0.63,1.26)	0.244
unvalidated	3	23.5%	1.31	(0.26,1.41)	0.520
**Study quality score**					
High (>6)	0				
Low (⩽6)	6	86.6%	0.76	(0.48,1.20)	0.240

Table S4. Subgroup analysis of SSB consumption and cancer mortality

Subgroup	No. of included studies	I^2	Random-effect model		
			OR	95%CI	P
**All studies**					
**Geographic location**					
European	1				
North America	7	69.7%	**1.10**	**(1.02,1.19)**	**0.013**
Asia	3	43.2%	0.99	(0.81,1.22)	0.952
**No. of cases**					
<500	3	81.8%	1.29	(0.66,2.50)	0.457
⩾500	7	31.3%	1.04	(0.98,1.10)	0.168
**Type of FFQ**					
validated	5	57.2%	**1.12**	**(1.02,1.23)**	**0.017**
unvalidated	6	58.8%	1.03	(0.95,1.12)	0.430
**Study quality score**					
High (>6)	6	45.1%	1.04	(0.99,1.10)	0.089
Low (⩽6)	5	71.2%	1.22	(0.93,1.60)	0.158
**Cohort studies**					
**Geographic location**					
European	1				
North America	6	59.0%	**1.08**	**(1.01,1.15)**	**0.022**
Asia	3	43.2%	0.99	(0.81,1.22)	0.952
**No. of cases**					
<500	2	85.9%	1.00	(0.36,2.83)	0.994
⩾500	7	31.3%	1.04	(0.98,1.10)	0.168
**Type of FFQ**					
validated	5	57.2%	**1.12**	**(1.02,1.23)**	**0.017**
unvalidated	5	5.9%	1.01	(0.97,1.06)	0.594
**Study quality score**					
High (>6)	6	45.1%	1.04	(0.99,1.10)	0.089
Low (⩽6)	4	59.2%	1.10	(0.86,1.43)	0.430

Table S5. The sensitivity analysis of the relationship between SSB intake and cancer risk

Excluded studies	No. of included studies	I^2	Random-effect model		
			OR	95%CI	P
**All studies**					
No	53	64.9%	**1.12**	**(1.06,1.20)**	**<0.001**
Chazelas2019	52	65.4%	**1.13**	**(1.06,1.20)**	**<0.001**
Makarem2018	52	65.6%	**1.13**	**(1.06,1.20)**	**<0.001**
Hodge2018	52	65.6%	**1.13**	**(1.06,1.20)**	**<0.001**
Li2017	52	65.4%	**1.12**	**(1.05,1.20)**	**<0.001**
Yassibas2012	52	63.8%	**1.12**	**(1.05,1.18)**	**<0.001**
Ren2010	52	63.2%	**1.13**	**(1.07,1.20)**	**<0.001**
Ibiebele2008	52	65.2%	**1.13**	**(1.06,1.20)**	**<0.001**
Mayne2006	52	60.8%	**1.13**	**(1.07,1.20)**	**<0.001**
Lagergren2006	52	65.6%	**1.12**	**(1.06,1.20)**	**<0.001**
Turati2015	52	65.6%	**1.12**	**(1.06,1.20)**	**<0.001**
Ros2011	52	65.5%	**1.13**	**(1.06,1.20)**	**<0.001**
Jensen1985	52	65.3%	**1.12**	**(1.06,1.19)**	**<0.001**
Jiang2008	52	65.3%	**1.12**	**(1.05,1.19)**	**<0.001**
Stefani2007	52	65.6%	**1.12**	**(1.06,1.19)**	**<0.001**
Bruemmer(males)2013	52	65.0%	**1.12**	**(1.05,1.19)**	**<0.001**
Holick2010	52	64.1%	**1.13**	**(1.07,1.20**	**<0.001**
Dubrow2012	52	65.0%	**1.13**	**(1.06,1.20)**	**<0.001**
Romanos2019	52	65.4%	**1.12**	**(1.06,1.19)**	**<0.001**
Marzbani2019	52	58.7%	**1.10**	**(1.04,1.16)**	**<0.001**
Chandran2014	52	65.6%	**1.12**	**(1.06,1.19)**	**<0.001**
Potischman2002	52	65.6%	**1.12**	**(1.06,1.19)**	**<0.001**
Stepien2016	52	64.8%	**1.13**	**(1.06,1.20)**	**<0.001**
Larsson2016	52	64.0%	**1.11**	**(1.05,1.18)**	**<0.001**
Rizk2019	52	64.5%	**1.12**	**(1.05,1.18)**	**<0.001**
Pacheco2019	52	65.5%	**1.12**	**(1.06,1.19)**	**<0.001**
Theodoratou2014	52	65.3%	**1.13**	**(1.06,1.20)**	**<0.001**
Mahfouz2014	52	63.8%	**1.12**	**(1.05,1.18)**	**<0.001**
Zhang2010	52	65.4%	**1.13**	**(1.06,1.20)**	**<0.001**
Fung2010	52	65.6%	**1.13**	**(1.06,1.20)**	**<0.001**
Bener2010	52	63.9%	**1.11**	**(1.05,1.18)**	**<0.001**
Mccullough2014	52	65.3%	**1.13**	**(1.06,1.20)**	**<0.001**
Schernhammer2012	52	65.1%	**1.12**	**(1.05,1.19)**	**<0.001**
King2013	52	65.6%	**1.12**	**(1.06,1.19)**	**<0.001**
King2013	52	65.2%	**1.12**	**(1.05,1.19)**	**<0.001**

Table S5. *(Continued)*

Excluded studies	No. of included studies	I^2	Random-effect model		
			OR	95%CI	P
Inoue-choi2013	52	63.4%	**1.11**	**(1.05,1.18)**	**<0.001**
Navarrete2016	52	65.2%	**1.13**	**(1.06,1.20)**	**<0.001**
Genkinger2012	52	65.4%	**1.12**	**(1.06,1.19)**	**<0.001**
Gallus2010	52	65.6%	**1.12**	**(1.06,1.19)**	**<0.001**
Mueller2010	52	64.6%	**1.12**	**(1.05,1.18)**	**<0.001**
Chan2009	52	65.5%	**1.13**	**(1.06,1.20)**	**<0.001**
Bao2008	52	64.2%	**1.13**	**(1.07,1.20)**	**<0.001**
Larsson2006	52	64.3%	**1.12**	**(1.051.18)**	**<0.001**
Schernhammer2005	52	65.6%	**1.12**	**(1.06,1.19)**	**<0.001**
Lyon1992	52	65.4%	**1.12**	**(105,1.19)**	**<0.001**
Miles2018	52	65.0%	**1.12**	**(1.05,1.19)**	**<0.001**
Darke2012	52	65.6%	**1.12**	**(1.06,1.19)**	**<0.001**
Sharpe2002	52	65.5%	**1.13**	**(1.06,1.20)**	**<0.001**
Ellison2000	52	65.5%	**1.12**	**(1.06,1.19)**	**<0.001**
Jain1998	52	65.0%	**1.13**	**(1.06,1.20)**	**<0.001**
Hu2009	52	65.3%	**1.12**	**(1.05,1.19)**	**<0.001**
lee2007	52	65.6%	**1.12**	**(1.06,1.19)**	**<0.001**
Talamini1990	52	65.4%	**1.12**	**(1.06,1.19)**	**<0.001**
Polesel2013	52	65.5%	**1.13**	**(1.06,1.20)**	**<0.001**
**Cohort studies**					
No	26	59.3%	**1.08**	**(1.01,1.15)**	**0.020**
Chazelas2019	25	60.8%	**1.09**	**(1.01,1.17)**	**0.025**
Makarem2018	25	60.9%	**1.08**	**(1.01,1.16)**	**0.020**
Hodge2018	25	60.9%	**1.08**	**(1.01,1.16)**	**0.026**
Ren2010	25	54.9%	**1.09**	**(1.03,1.17)**	**0.006**
Ros2011	25	60.9%	**1.08**	**(1.01,1.16)**	**0.020**
Holick2010	25	57.0%	**1.09**	**(1.02,1.16)**	**0.007**
Dubrow2012	25	59.7%	**1.09**	**(1.02,1.16)**	**0.012**
Romanos2019	25	60.4%	**1.08**	**(1.01,1.15)**	**0.026**
Stepien2019	25	59.7%	**1.09**	**(1.02,1.17)**	**0.016**
Larsson2016	25	55.8%	**1.09**	**(1.00,1.14)**	**0.040**
Pacheco2019	25	60.7%	**1.08**	**(1.01,1.15)**	**0.027**
Zhang2010	25	60.6%	**1.08**	**(1.01,1.16)**	**0.016**
Fung2010	25	60.7%	**1.08**	**(1.01,1.16)**	**0.029**
Mccullough2014	25	60.4%	**1.09**	**(1.02,1.16)**	**0.016**
Schemhammer2012	25	59.2%	**1.07**	**(1.00,1.15)**	**0.038**
Inoue-choi2013	25	53.7%	**1.06**	**(1.00,1.13)**	**0.051**

Table S5. *(Continued)*

Excluded studies	No. of included studies	I^2	Random-effect model		
			OR	95%CI	P
Nararrete2016	25	60%	**1.09**	**(1.02,1.16)**	**0.013**
Genkinger2012	25	60.1%	**1.08**	**(1.01,1.15)**	**0.033**
Mueller2010	25	57.9%	**1.07**	**(1.01,1.14)**	**0.033**
Bao2008	25	57.5%	**1.09**	**(1.02,1.17)**	**0.008**
Larsson2006	25	56.9%	**1.07**	**(1.00,1.14)**	**0.036**
Schemhammer2005	25	60.8%	**1.08**	**(1.01,1.15)**	**0.024**
Miles2018	25	58.3%	**1.07**	**(1.00,1.15)**	**0.041**
Darke2012	25	60.7%	**1.08**	**(1.01,1.15)**	**0.028**
Ellison2000	25	60.6%	**1.08**	**(1.01,1.15)**	**0.025**
Lee2007	25	60.8%	**1.08**	**(1.01,1.16)**	**0.026**
**Case-control studies**					
No	27	68.5%	**1.20**	**(1.06,1.35)**	**0.003**
Li2017	26	69.5%	**1.20**	**(1.06,1.36)**	**0.005**
Yassibas2012	26	67.0%	**1.18**	**(1.05,1.32)**	**0.006**
Ibiebele2008	26	69.0%	**1.21**	**(1.07,1.36)**	**0.002**
Mayne2006	26	58.8%	**1.23**	**(1.10,1.36)**	**0.000**
Lagergren2006	26	69.7%	**1.20**	**(1.06,1.36)**	**0.004**
Turati2015	26	69.6%	**1.21**	**(1.07,1.37)**	**0.003**
Jensen1985	26	69.3%	**1.19**	**(1.05,1.34)**	**0.005**
Jiang2008	26	69.4%	**1.19**	**(1.05,1.35)**	**0.005**
Stefani2007	26	69.7%	**1.20**	**(1.06,1.36)**	**0.003**
Bruemmen2013	26	68.9%	**1.18**	**(1.05,1.34)**	**0.006**
Marzbani2019	26	58.0%	**1.14**	**(1.03,1.27)**	**0.014**
Chandran2014	26	69.7%	**1.20**	**(1.06,1.37)**	**0.004**
Patischman2002	26	69.7%	**1.20**	**(1.06,1.36)**	**0.003**
Rizk2019	26	68.1%	**1.18**	**(1.05,1.33)**	**0.006**
Theodoratou2014	26	69.6%	**1.22**	**(1.05,1.41)**	**0.008**
Mahfouz2014	26	66.8%	**1.18**	**(1.05,1.32)**	**0.005**
Bener2010	26	67.5%	**1.18**	**(1.04,1.33)**	**0.008**
King2013	26	69.7%	**1.20**	**(1.06,1.36)**	**0.003**
King2013	26	69.2%	**1.19**	**(1.05,1.34)**	**0.005**
Gallus2010	26	69.6%	**1.21**	**(1.07,1.37)**	**0.003**
Chan2009	26	69.5%	**1.21**	**(1.07,1.37)**	**0.003**
Lyon1992	26	69.5%	**1.19**	**(1.06,1.35)**	**0.005**
Sharpe2002	26	69.5%	**1.21**	**(1.07,1.37)**	**0.003**
Jain1998	26	68.5%	**1.22**	**(1.08,1.37)**	**0.001**

Table S5. *(Continued)*

Excluded studies	No. of included studies	I^2	Random-effect model		
			OR	95%CI	P
Hu2009	26	69.5%	**1.20**	**(1.06,1.36)**	**0.005**
Talamini1990	26	69.4%	**1.20**	**(1.07,1.36)**	**0.002**
Polesel2013	26	69.4%	**1.21**	**(1.07,1.37)**	**0.002**

Table S6. The sensitivity analysis of the relationship between fruit juice intake and cancer risk

Excluded studies	No. of included studies	I^2	Random-effect model		
			OR	95%CI	P
**All studies**					
**No**	17	68.4%	1.05	(0.95,1.16)	0.338
Chazelas2019	16	69.0%	1.04	(0.93,1.15)	0.514
Stepien2016	16	69.2%	1.04	(0.92,1.18)	0.501
Makarem2018	16	67.8%	1.03	(0.93,1.14)	0.543
Pourfarzi2009	16	70.0%	1.04	(0.94,1.15)	0.407
Jiang2008	16	70.2%	1.05	(0.95,1.16)	0.311
Farvid2016	16	70.3%	1.05	(0.94,1.17)	0.373
Mahfouz2014	16	42.0%	1.08	(1.00,1.16)	**0.044**
Annema2011	16	66.9%	1.03	(0.93,1.13)	0.574
Inoue-choi2013	16	70.0%	1.04	(0.94,1.15)	0.438
Mueller2010	16	70.0%	1.04	(0.94,1.15)	0.408
Nothling2007	16	70.3%	1.05	(0.94,1.16)	0.395
Chan2005	16	66.4%	1.07	(0.97,1.18)	0.149
Miles2018	16	70.3%	1.04	(0.94,1.16)	0.448
Darke2012	16	70.2%	1.05	(0.95,1.17)	0.337
Lee2007	16	70.3%	1.04	(0.94,1.16)	0.409
Rashidkhani2004	16	69.0%	1.04	(0.94,1.14)	0.474
Sanchez2003	16	68.5	1.06	(0.97,1.17)	0.211
**Cohort studies**					
No	11	7.2%	**1.06**	**(1.01,1.11)**	**0.013**
Chazelas2019	10	0%	1.04	(1.00,1.08)	0.056
Stepien2016	10	0%	**1.10**	**(1.04,1.18)**	**0.002**
Makarem2018	10	0%	**1.04**	**(1.00,1.09)**	**0.028**
Farvid2016	10	15.5%	**1.08**	**(1.02,1.14)**	**0.012**
Inoue-choi2013	10	11.9%	**1.06**	**(1.01,1.12)**	**0.019**
Mueller2010	10	11.6%	**1.06**	**(1.01,1.12)**	**0.016**
Nothing2007	10	16.1%	**1.07**	**(1.01,1.13)**	**0.016**
Miles2018	10	15.8%	**1.07**	**(1.01,1.14)**	**0.021**
Darke2012	10	13.8%	**1.07**	**(1.01,1.13)**	**0.010**
Lee2007	10	15.5%	**1.07**	**(1.01,1.13)**	**0.017**
Rashidkhani2004	10	0%	**1.05**	**(1.01,1.09)**	**0.019**
**Case-control studies**					
No	6	86.6%	0.76	(0.48,1.20)	0.240
Pourfarzi2009	5	88.9%	0.68	(0.40,1.16)	0.158

Table S6. *(Continued)*

Excluded studies	No. of included studies	I^2	Random-effect model		
			OR	95%CI	P
Jiang2008	5	89.3%	0.73	(0.43,1.25)	0.258
Mahfouz2014	5	73.1%	0.96	(0.68,1.34)	0.802
Annema2011	5	79.7%	0.66	(0.41,1.07)	0.090
Chan2005	5	88.1%	0.76	(0.41,1.36)	0.360
Sanchez2003	5	88.8%	0.76	(0.43,1.33)	0.335

Table S7. The sensitivity analysis of the relationship between SSB intake and cancer mortality

Excluded studies	No. of included studies	I^2	Random-effect model		
			OR	95%CI	P
**All studies**					
**No**	11	61.8%	**1.07**	**(1.01,1.14)**	**0.029**
Zhang2020	10	64.8%	**1.07**	**(1.01,1.15)**	**0.033**
Tasevska2014	10	64.2%	**1.10**	**(1.01,1.19)**	**0.033**
Odegaard2015	10	60.1%	**1.09**	**(1.02,1.16)**	**0.009**
Mullee2019	10	64.1%	**1.08**	**(1.01,1.16)**	**0.032**
Malik2019	10	63.9%	1.07	(0.99,1.16)	0.094
Khan2004	10	64.8%	**1.07**	**(1.00,1.14)**	**0.036**
Guercio2018	10	60.7%	**1.08**	**(1.01,1.14)**	**0.016**
Fuchs2014	10	60.5%	**1.06**	**(1.00,1.13)**	**0.045**
Barrington2016	10	61.0%	1.06	(0.99,1.13)	0.071
Miles2016	10	50.9%	**1.06**	**(1.00,1.12)**	**0.046**
Fung2014	10	63.3%	1.07	(1.00,1.15)	0.060
**Cohort studies**					
No	10	50.9%	1.06	**(1.00,1.12)**	**0.046**
Zhang2020	9	55.0%	1.06	(1.00,1.12)	0.054
Tasevska2014	9	53.9%	1.10	(1.04,1.18)	0.061
Odegaard2015	9	46.6%	**1.07**	**(1.01,1.12)**	**0.014**
Mullee2019	9	54.1%	1.06	(1.00,1.13)	0.055
Malik2019	9	51.4%	1.05	(0.98,1.12)	0.184
Khan2004	9	53.7%	1.05	(1.00,1.11)	0.052
Guercio2018	9	47.5%	**1.06**	**(1.01,1.12)**	**0.025**
Fuchs2014	9	46.7%	1.05	(1.00,1.11)	0.057
Barrington2016	9	47.5%	1.05	(0.99,1.10)	0.101
Fung2014	10	52.0%	1.05	(0.99,1.11)	0.107

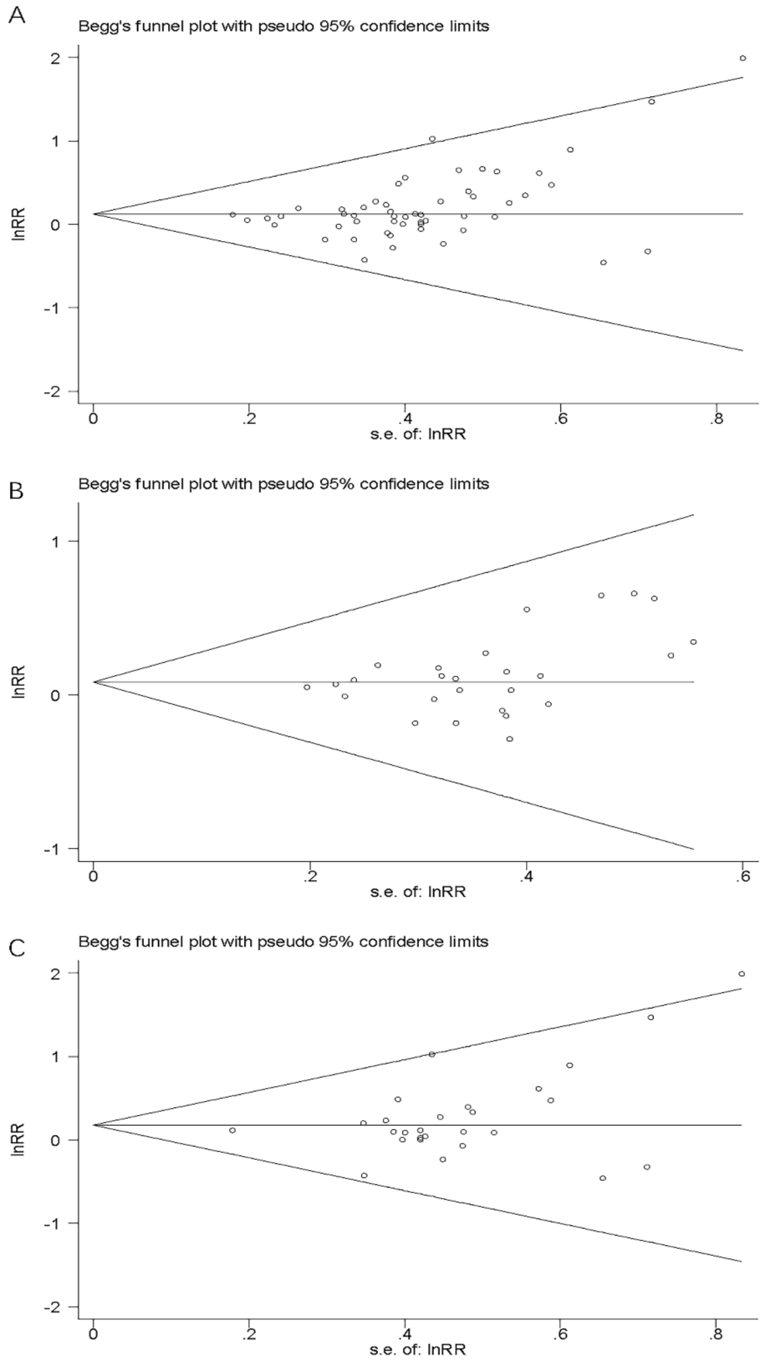

Fig.S1 Funnel plot for publication bias for SSB consumption and cancer risk by all studies (A), the cohort studies (B), and the case-control studies(C).

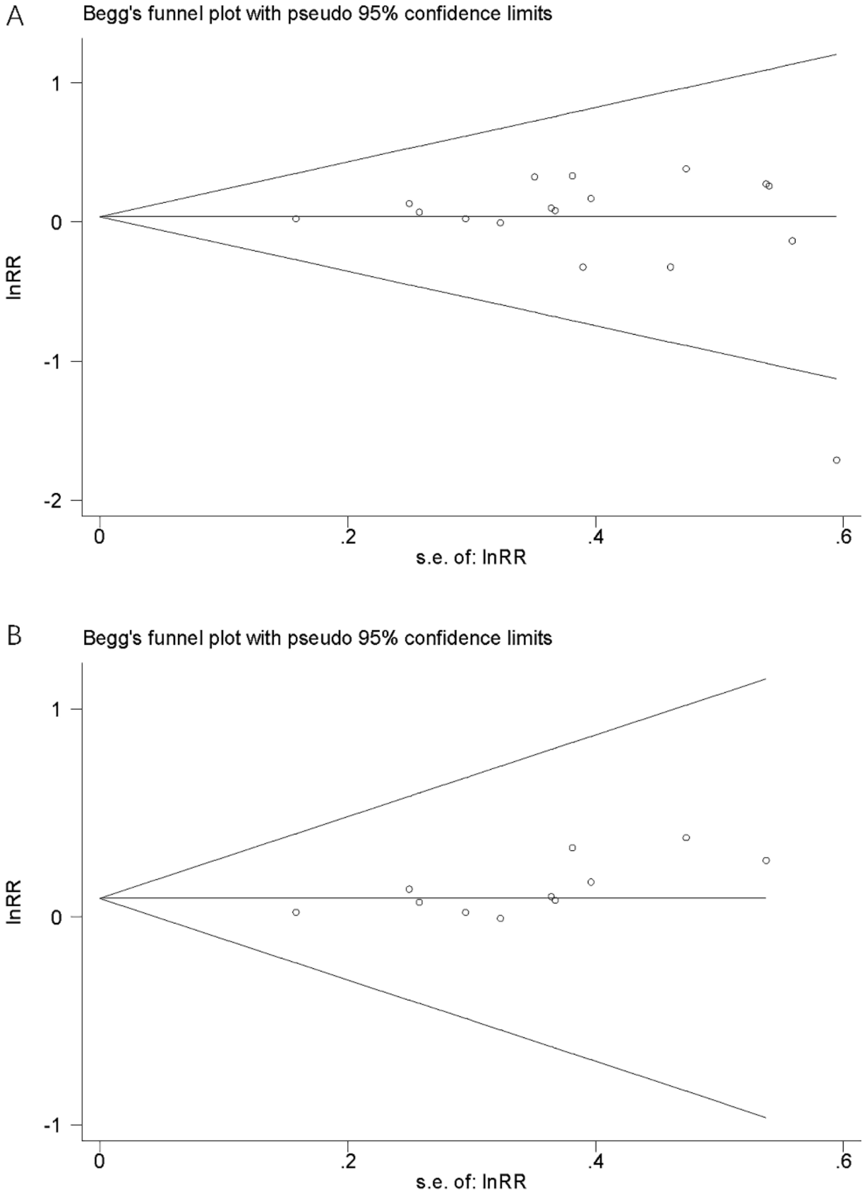

Fig.S2 Funnel plot for publication bias for fruit juice consumption and cancer risk by all studies (A), and the cohort studies (B).

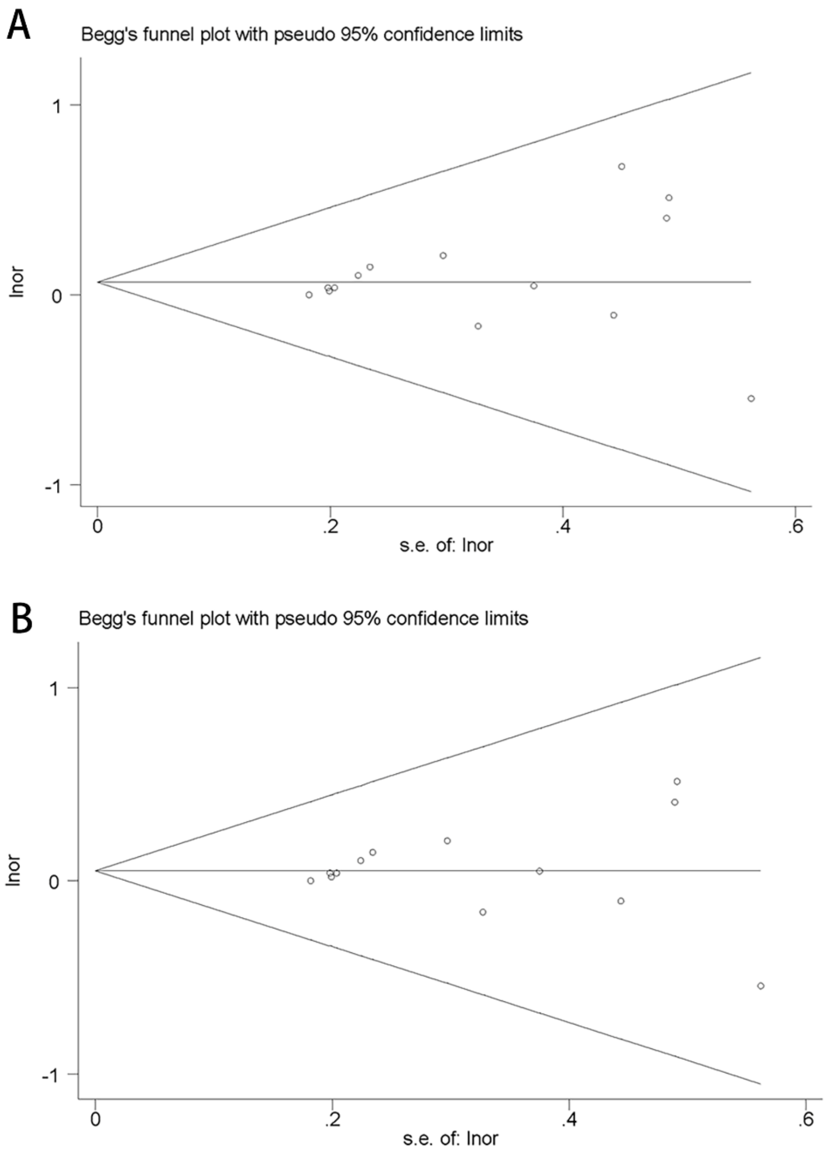

Fig.S3 Funnel plot for publication bias for SSB consumption and cancer mortality by overall studies (A), and the cohort studies (B).
